# Navigating the microarray landscape: a comprehensive review of feature selection techniques and their applications

**DOI:** 10.3389/fdata.2025.1624507

**Published:** 2025-07-10

**Authors:** Fangling Wang, Azlan Mohd Zain, Yanjie Ren, Mahadi Bahari, Azurah A. Samah, Zuraini Binti Ali Shah, Norfadzlan Bin Yusup, Rozita Abdul Jalil, Azizah Mohamad, Nurulhuda Firdaus Mohd Azmi

**Affiliations:** ^1^Faculty of Computing, Universiti Teknologi Malaysia, Skudai, Johor, Malaysia; ^2^Hebei Institute of Mechanical and Electrical Technology, Xingtai, China; ^3^Faculty of Management, Universiti Teknologi Malaysia, Skudai, Johor, Malaysia; ^4^Faculty of Computer Science and Information Technology, Universiti Malaysia Sarawak, Kota Samarahan, Malaysia; ^5^Department of Software Engineering, Faculty of Computer Science and Information Technology, Universiti Tun Hussein Onn Malaysia, Parit Raja, Johor, Malaysia; ^6^Faculty of Computing, Universiti Malaysia Pahang Al-Sultan Abdullah, Kuantan, Pahang, Malaysia; ^7^Faculty of Artificial Intelligence, Universiti Teknologi Malaysia, Skudai, Johor, Malaysia

**Keywords:** cancer classification, feature selection, microarray data, machine learning, gene expression analysis

## Abstract

This review systematically summarizes recent advances in microarray feature selection techniques and their applications in biomedical research. It addresses the challenges posed by the high dimensionality and noise of microarray data, aiming to integrate the strengths and limitations of various methods while exploring their applicability across different scenarios. By identifying gaps in current research, highlighting underexplored areas, and proposing clear directions for future studies, this review seeks to inspire academics to develop novel techniques and applications. Furthermore, it provides a comprehensive evaluation of feature selection methods, offering both a theoretical foundation and practical guidance to help researchers select the most suitable approaches for their specific research questions. Emphasizing the importance of interdisciplinary collaboration, the study underscores the potential of feature selection in transformative applications such as personalized medicine, cancer diagnosis, and drug discovery. Through this review, not only does it provide in-depth theoretical support for the academic community, but also practical guidance for the practical field, which significantly contributes to the overall improvement of microarray data analysis technology.

## 1 Introduction

The microarray is a powerful biotechnological tool that allows for the simultaneous evaluation of the expression levels of multiple genes (Joseph and Sandoval, 2023). This technique involves immobilizing numerous nucleic acid probes onto a solid surface, such as a glass slide or a silicon chip, which are designed to specifically interact with their corresponding RNA or DNA sequences (Wang et al., 2023a). Through the examination of probe-target interactions, scientists can determine the expression levels of each gene in the sample. Due to its versatility, microarray technology finds broad applications in the study of gene expression mechanisms, identification of biomarkers, disease diagnosis, and pharmaceutical development. On the other hand, the expression levels of thousands of genes can be studied simultaneously in microarray experiments, which are a crucial aspect of modern molecular biology (Maolmhuaidh et al., 2023). However, the resulting data can be challenging to analyze due to their high dimensionality and small sample size. This complexity often leads to inaccurate results and unreliable conclusions when traditional statistical methods and machine learning algorithms are applied directly (Prajapati et al., 2023b). To address these issues, microarray feature selection techniques are employed to identify the most informative gene features, thereby reducing the complexity of the data and improving its interpretability.

Despite the advantages of microarray datasets, excessively high dimensions can have several negative effects on model performance in microarray data analysis, including overfitting, increasing computational costs, and poor interpretability of results. To combat these issues, various methods are used when dealing with microarray datasets that contain too many dimensions. Commonly used methods include feature selection and feature extraction (Labory et al., 2024). Compared to feature extraction, feature selection retains biological significance and interpretability by filtering the most important original features, and usually has a lower computational overhead (Pudjihartono et al., 2022). Feature selection has significant advantages over feature extraction in the downscaling process of microarray data (Pirch et al., 2021). First, feature selection preserves the original gene characteristics and thus results are more interpretable, which enables researchers to directly correlate selected genes with specific biological processes or disease mechanisms, providing clear guidance for biological research and clinical applications. Second, the high biological relevance of feature selection helps identify potential biomarkers and provide insight into the molecular mechanisms of disease. In addition, feature selection methods are often computationally more efficient, especially when dealing with large-scale microarray data, and many filtering methods based on statistical tests can quickly and efficiently screen out important features. By reducing the number of features, feature selection also reduces the complexity of the model, thereby minimizing the risk of overfitting, which is particularly important for high-dimensional microarray data with a limited number of samples. Finally, since feature selection preserves the original feature structure, the model can be trained and predicted directly using these features, avoiding complex transformation or preprocessing steps. As a result, feature selection shows clear advantages in scenarios that require high interpretability, direct biological relevance, and computational efficiency.

Microarray feature selection is an essential step in the analysis of gene expression data. It helps streamline the data, making it more accessible for study and providing actionable insights for researchers. Focusing on the most informative features through feature selection can not only improve the quality and interpretability of the data, but also establish a foundation for the development of the precise predictive model. However, inadequate feature selection can lead to several challenges in the analysis of microarray data, such as increased risk of overfitting, inefficient use of computational resources, and reduced clarity of data interpretation. Overcoming these issues can enable researchers to gain a deeper understanding of data and advance biomedical research. In recent developments, many studies have identified problems in existing microarrays and proposed methods to solve these problems. For example, Fadhil and Abdulazeez (2024) summarized the application of deep learning methods to overcome the high-dimensionality problem of microarray datasets. They explored how deep learning methods can be applied in the complex research field of cancer classification. Osama et al. (2023) summarized preprocessing methods for microarray datasets and discussed different feature selection methods based on machine learning. In contrast, Hambali et al. (2020) failed to provide an application of feature selection in their summary of different feature selection techniques. Given these drawbacks, there has been a lack of comprehensive summaries that cover the entire process of microarray feature selection, this study covers a wider range of research areas than previous reviews, especially summarizing research results in recent years, allowing researchers to better understand research trends.

To bridge this gap, this paper aims to explore dataset-specific feature selection methods and summarize the advantages and disadvantages of each category of methods. Additionally, this article will discuss the various application areas of microarrays. In preparation for this paper, the keyword 'microarray feature selection' was used to search for articles published after 2019 on Google Scholar. This review will first provide an overview of the basic concepts of microarray technology. Next, various methods of microarray feature selection will be compared and analyzed, different application fields of microarray feature selection will be summarized, and technical challenges and potential future research directions in this field will be evaluated. The following chapters will cover key aspects of microarray feature selection. Section 2 will give a detailed overview of the relevant concepts of microarray feature selection. Section 3 will comprehensively review the existing literature and evaluate the advantages and disadvantages of various feature selection methods. Section 4 will focus on the practical applications of microarray feature selection in different fields. The final section will explore potential issues and predict future development directions, aiming to provide valuable guidance and insights for future research.

## 2 Microarray feature selection process

This section focuses on basic concepts and terminology related to microarray feature selection, providing an in-depth look at the complexity of microarray datasets, design principles, and the various advantages and disadvantages of different feature selection methods. Furthermore, it will be explored how to evaluate and compare the effectiveness of these methods and how to select the most appropriate subset of features to achieve accurate model results.

### 2.1 Introduction

**Figure 1:**
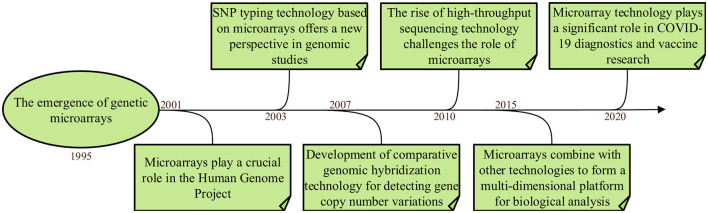
The development history of microarray.

Microarray technology dates back to the late 1990s and early 2000s and is designed to measure the expression levels of numerous genes simultaneously (Moses and Pachter, 2022). As microarray technology continues to mature, the fields of molecular biology, bioinformatics and statistical analysis have also made great progress (Singh et al., 2023). These advances ensure that microarray technology remains an indispensable tool for systems biologists and clinical researchers, driving discoveries and improving patient care (Vatansever et al., 2021). Figure 1 shows The development of microarrays in recent years. Researchers can use high-throughput microarray technology to simultaneously analyze the expression levels of thousands of genes or detect specific DNA sequences, which allows researchers to delve deeper into a gene's activity under specific circumstances. The technology can also be used for gene expression analysis, genotyping, drug discovery and disease diagnosis.

In the analysis of high-dimensional microarray data, the choice of feature selection methods is critical to control the risk of overfitting. Microarray data are usually of extremely high dimensionality but with a limited number of samples, resulting in a model that is prone to overfitting on the training data. This risk can be effectively reduced by choosing an appropriate feature selection method. First, feature selection removes redundant and noisy features and reduces model complexity, which is critical to minimizing overfitting. Controlling the number of features selected is equally critical; too many features may cause the model to capture random fluctuations instead of the true signal. In addition, a robust feature selection method improves the reliability of the selected features and avoids instability due to small variations in the data, which further reduces the likelihood of overfitting. Choosing a feature selection method that matches the complexity of the model ensures that the selected feature set best fits the current model. Finally, embedding cross-validation into the feature selection process can more accurately assess the contribution of features to model performance and avoid features that are only valid for the training data, thus effectively reducing the risk of overfitting.

The class imbalance problem in microarray datasets can be effectively addressed through feature selection, and the key is to employ multiple strategies to enhance the recognition of minority classes. First, prioritizing features that can significantly differentiate between minority and majority classes ensures that the model is more likely to capture signals from minority classes. Second, class weights are introduced into the feature selection process so that features of minority class samples are given higher importance in the selection. In addition, balancing the dataset before feature selection through undersampling or oversampling techniques prevents the majority class from dominating the feature selection process, resulting in a more representative feature set.

This paper will examine specific feature selection methods in more detail, discussing their theoretical bases, practical applications, and the challenges associated with implementing these methods in different research contexts. This discussion will provide a comprehensive understanding of how microarray feature selection is integral to refining data analysis and ensuring the reliability of research outcomes in the field of genomics.

**Figure 2:**
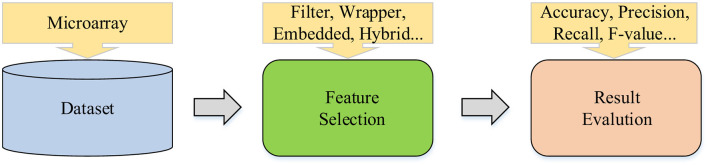
Microarray feature selection main concept.

Based on the overview provided previously, the main concepts of microarray feature selection can be divided into three key parts, as shown in Figure 2. This visual framework helps succinctly organize various aspects of feature selection into a coherent structure, thereby promoting deeper understanding. Next, we will delve into the related concepts of these three parts. Each component plays a unique role throughout the feature selection process, covering everything from initial data preparation to the final selection of features that best predict the outcome of interest.

### 2.2 Microarray dataset

Microarray technology, a pivotal tool in genomic research, enables the high-throughput analysis of gene expression across numerous conditions and diseases (Yang et al., 2020). Within the realm of binary classification, several classic datasets are frequently employed, each specific to particular types of cancer or disease states. For instance, the Colon Cancer dataset includes gene expression profiles from colon tissue and is used to study colorectal cancer. This dataset helps in identifying genes or patterns associated with different stages or types of colon cancer, thereby aiding in diagnostics and potential treatment strategies (Shafi et al., 2020). Similarly, the leukemia dataset provides gene expression data specifically related to leukemia, a type of blood cancer. Including samples from various subtypes of leukemia, this dataset allows researchers to delve into the molecular characteristics of the disease and identify potential biomarkers for diagnosis and treatment. Additionally, the prostate dataset focuses on prostate cancer, a prevalent condition among men. It contains gene expression profiles associated with prostate tissue or cells to identify markers that can differentiate benign from malignant prostate disease or enhance our understanding of disease progression. Another key dataset is the DLBCL dataset, which stands for diffuse large B-cell lymphoma, one of the most common non-Hodgkin lymphomas. It includes gene expression data from lymphoma tissues, helping researchers to identify genetic markers or patterns associated with different DLBCL subtypes and treatment responses (Shukla and Tripathi, 2020). What's more, the CNS dataset involves various molecular data related to diseases affecting the central nervous system. This dataset includes gene expression profiles from conditions such as brain tumors and neurological disorders, enabling researchers to understand the molecular signatures associated with CNS disorders (Sánchez-Maroño et al., 2019).

For multi-classification datasets, this study also summarizes commonly used datasets, which are equally important in genomic research. The SRBCT dataset involves gene expression profiling of small round blue cell tumors. The dataset includes four categories of tumors and is commonly used to distinguish them and identify specific genetic markers associated with each subtype (Sahu and Dash, 2023). Likewise, the Lung Cancer (Harvard) dataset focusing on lung cancer is another great resource (Karthika et al., 2023). Organized into five categories, the dataset helps identify genetic patterns that distinguish various subtypes or stages of lung cancer, thereby aiding diagnostic and treatment strategies. Additionally, the Leukemia2 dataset contains three categories that help researchers understand the molecular differences between leukemia subtypes and assist in identifying biomarkers for accurate diagnosis or targeted therapy (Rupapara et al., 2022). Additionally, the 9Tumor and Brain Tumor1 datasets provide valuable insights into molecular variations between different tumor types or subtypes in different tissues or organs, aiding in classification and providing potential therapeutic insights (Zhu et al., 2023).

**Figure 3:**
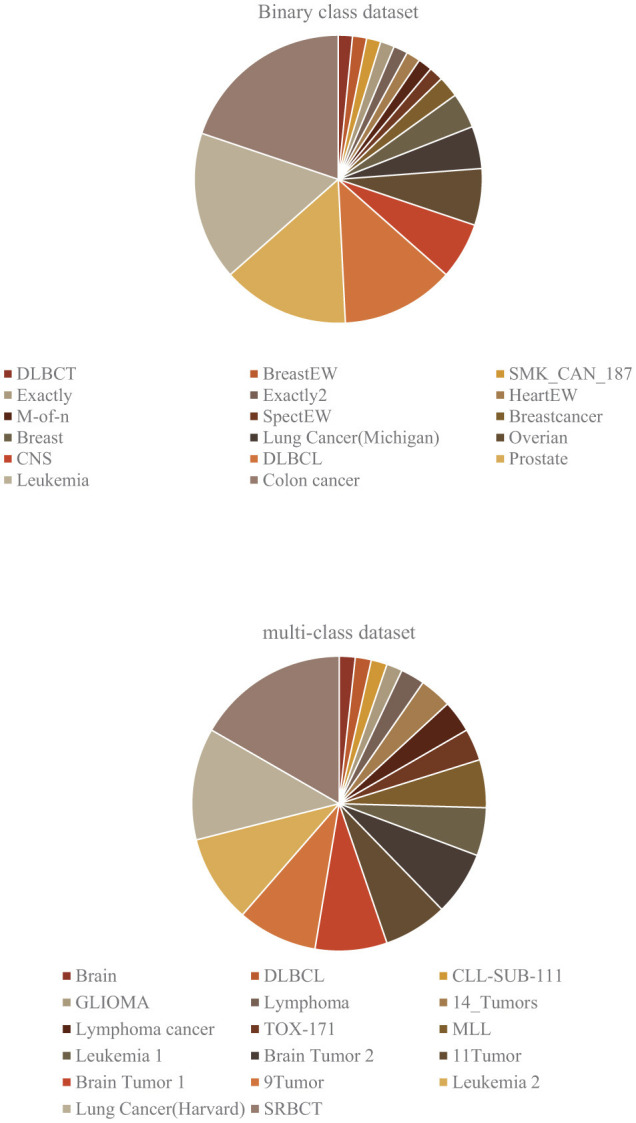
Proportion of commonly used datasets.

In summary, these microarray datasets, encompassing both binary and multi-classification data, serve as invaluable resources for researchers across numerous fields. By exploring and analyzing these datasets, scientists can uncover crucial insights and advancements in areas such as cancer research and neurological disorders. This paper provides a comprehensive analysis of datasets used in various articles highlighted the most frequently employed datasets in both binary and multi-class classifications, as detailed in Tables 1, 2. This review not only underscores the importance of these datasets but also reflects ongoing efforts to address the challenges associated with microarray data analysis. Figure 3 provides a visual representation of the proportion of datasets used, further illustrating the critical role these datasets play in advancing our understanding of complex biological processes and diseases.

**Table 1:** Binary class dataset.

**Dataset**	**Sample**	**Feature**	**Where used**
DLBCT	77	7,070	Tavasoli et al., 2021; Zhou et al., 2021
BreastEW	30	569	Chatterjee et al., 2020; Guha et al., 2020
SMK_CAN 187	187	19,993	Climente-González et al., 2019; Nematzadeh et al., 2019
Breast	97	24,481	Baliarsingh et al., 2020; Pirgazi et al., 2019
Breast cancer	9	699	Chatterjee et al., 2020; Das et al., 2022; Guha et al., 2020
Lung Cancer (Michigan)	96	7,129	Han et al., 2021; Jain and Singh, 2021; Kang et al., 2019; Pirgazi et al., 2019
Ovarian	253	15,154	Baliarsingh et al., 2020; Ganesh et al., 2023; Jain and Singh, 2021; Kang et al., 2019
CNS	60	7,129	Kang et al., 2019; Nematzadeh et al., 2019; Peng et al., 2021; Pirgazi et al., 2019; Saberi-Movahed et al., 2022
Prostate	102	12,600	Guha et al., 2020; Jain and Singh, 2021; Mandal et al., 2021; Peng et al., 2021; Pirgazi et al., 2019; Shukla et al., 2019b,c; Tatwani and Kumar, 2019; Tavasoli et al., 2021; Zhou et al., 2021
DLBCL	77	7,129	Chatterjee et al., 2020; Dhal and Azad, 2021; Guha et al., 2020; Han et al., 2021; Hosseini and Moattar, 2019; Kang et al., 2019; Mandal et al., 2021; Peng et al., 2021; Pirgazi et al., 2019; Saberi-Movahed et al., 2022; Shukla et al., 2019b,c
Leukemia	72	7,129	Abdel-Basset et al., 2021; Baliarsingh et al., 2020; Das et al., 2022; Guha et al., 2020; Jain and Singh, 2021; Mandal et al., 2021; Peng et al., 2021; Pirgazi et al., 2019; Qiu, 2019; Saberi-Movahed et al., 2022; Tatwani and Kumar, 2019; Tavasoli et al., 2021
Colon	62	2,000	Abdel-Basset et al., 2021; Baliarsingh et al., 2020; Das et al., 2022; Ganesh et al., 2023; Guha et al., 2020; Han et al., 2021; Hosseini and Moattar, 2019; Jain and Singh, 2021; Nematzadeh et al., 2019; Peng et al., 2021; Pirgazi et al., 2019; Qiu, 2019; Saberi-Movahed et al., 2022; Shukla et al., 2019b,c; Tatwani and Kumar, 2019; Tavasoli et al., 2021

**Table 2:** Multi-class dataset.

**Dataset**	**Sample**	**Feature**	**Class**	**Where used**
Leukemia 1	72	5,327	3	Shukla et al., 2019b; Sun et al., 2019; Zhou et al., 2021
Leukemia 2	72	11,225	3	Chatterjee et al., 2020; Shukla et al., 2019b; Zhou et al., 2021
MLL	72	12,582	3	Guha et al., 2020; Kang et al., 2019
SRBCT	83	2,308	4	Chatterjee et al., 2020; Dhal and Azad, 2021; Guha et al., 2020; Shukla et al., 2019b,c
GLIOMA	50	4,434	4	Climente-González et al., 2019; Saberi-Movahed et al., 2022
TOX-171	171	5,748	4	Climente-González et al., 2019; Kang et al., 2019; Saberi-Movahed et al., 2022
Brain Tumor 2	50	10,367	4	Dhal and Azad, 2021; Sun et al., 2019; Zhou et al., 2021
Brain Tumor 1	90	5,920	5	Dhal and Azad, 2021; Shukla et al., 2019b; Zhou et al., 2021
Lung(H)	203	12,600	5	Dhal and Azad, 2021; Jain and Singh, 2021; Shukla et al., 2019b; Zhou et al., 2021
9Tumor	60	5,726	9	Dhal and Azad, 2021; Shukla et al., 2019b; Zhou et al., 2021
Lymphoma	62	4,026	9	Kang et al., 2019; Peng et al., 2021; Saberi-Movahed et al., 2022
11Tumor	174	12,533	11	Dhal and Azad, 2021; Shukla et al., 2019b; Zhou et al., 2021

### 2.3 Feature selection method

Despite the power of microarray technology in analyzing gene expression and other biological processes, it still faces several challenges. These include the complexity of data analysis, high costs, sensitivity and dynamic range issues, and the need for high-quality biological samples. In addition, microarray data often exhibit characteristics such as high dimensionality and small sample sizes, which pose additional challenges such as noise and outlier issues (Hamraz et al., 2023). Feature selection is the main approach to this problem, with the goal of selecting a subset of the most important and useful features from a larger set of attributes or variables (Dhal and Azad, 2022). This process is particularly important in microarray data analysis because it identifies features that represent gene or protein expression levels and can better enhance data analysis. By identifying and retaining only the most important features, feature selection can greatly improve prediction accuracy and generalization capabilities, especially when dealing with limited sample data. In microarray analysis, this approach helps pinpoint genes associated with specific biological processes or disease states, providing valuable insights for interpretation and discovery of potential therapeutic targets.

Given the large number of genes typically present in microarray data, it is often the case that only a subset of these genes are relevant to the specific biological process or disease being studied (Jovic et al., 2022). The challenges of microarray data analysis are amplified by the presence of redundant features, which significantly increases computational complexity and the risk of overfitting. For example, for a dataset containing *N* features, the number of potential feature subsets is up to 2^*N*^ (Singh and Singh, 2021). This high dimensionality increases the risk of overfitting and highlights the urgent need to select a high-quality feature subset. Without effective feature selection, analysis can produce inaccurate results and lead to unnecessarily complex models. Feature selection simplifies analysis by reducing data dimensionality, which not only makes analysis more effective and efficient, but also improves prediction accuracy, increases interpretability, reduces the risk of overfitting, and improves computational efficiency.

Therefore, feature selection is a critical pre-processing step before applying machine learning algorithms to simplify data by eliminating irrelevant or redundant features. This improves model accuracy, reduces computational load, and produces results that are easier to interpret. In microarray analysis, the dimensionality of the data is very high, so how to obtain the optimal feature subset is particularly important (Lee et al., 2021). A good feature subset will significantly affect the performance and interpretability of the model (Yun et al., 2023). Each step in the feature selection process is closely linked, so it is crucial to design and execute these steps carefully. The feature selection process in microarray analysis begins with an initial subset search to create an initial subset of features that is evaluated and compared to previously considered subsets. If a new subset is found to be more suitable under the given evaluation criteria, this subset is retained. This iterative process continues until a predefined stopping condition is met, marking the end of the feature selection process. The selected feature subset is then used to verify the effectiveness of the feature selection method. Among the most commonly used methods for feature selection in microarrays are filter, wrapper, embedded, and other methods.

The filter feature selection method is characterized by simplicity and effectiveness. It filters features to eliminate those features that have the least impact on the target variable. This is usually achieved by setting a threshold or selecting the top k features based on statistical significance, this method can minimize the computational overhead. Evaluate the importance of each feature by calculating indicators such as information gain (IG), mutual information (MI), chi-square test, correlation coefficient, minimum redundancy, maximum correlation or Fisher score of the feature (Gong et al., 2022), rank these features according to importance and select those with the highest importance.

The wrapper method is a feature selection technique that directly links the evaluation of feature subsets to the performance of a machine learning model (Effrosynidis and Arampatzis, 2021). Unlike filter methods that rely on general statistical measures, wrapper methods are inherently more complex, as they involve training the model multiple times with different subsets of features and determining the most effective combination of features that enhance model performance through an iterative process. Wrapper methods can be divided into three core steps: the first step involves generating various feature subsets; the second step is the evaluation phase, where each subset is used to train the model to assess its performance; the final step involves selecting the feature subset that meets the criteria best, thereby effectively optimizing the model's predictive accuracy.

In the literature, the application of wrapper methods is usually a combination of intelligent optimization algorithms to search as many possible feature subsets as possible, and classifiers to identify those features that maximize the performance of the classifier. Subset. The role of the classifier is crucial as it evaluates the quality of each feature subset in terms of prediction accuracy. Through this collaborative interaction, wrapper methods exploit iterative refinement of feature subsets, aiming to arrive at a near-optimal set. Specifically, the effectiveness of wrapper methods depends on their ability to fine-tune the feature selection process through continuous feedback between subset evaluation and model training stages. This feedback is crucial to obtain the most informative subset of genes, thereby ensuring that the final subset of features is selected to be the best suited for the specific prediction task of the model.

The embedded feature selection is an important component in machine learning and data analysis due to its integration in the model training process. Unlike other techniques that operate independently of the training process, embedded methods make feature selection an inherent part of model learning. This integration allows the method to evaluate feature importance directly through the learning algorithm itself. During the training phase, the embedded method automatically weighs the relevance of each feature, allowing the model to focus on those features that are most critical for the prediction task. This inherent capability makes the model more effective in reducing the risk of overfitting and improving overall prediction accuracy by focusing on the most relevant features. The efficiency of the embedded approach is further demonstrated by its simplified feature selection process, which is directly embedded into the model's training algorithm, which simplifies the entire process and helps develop more efficient and effective machine learning models.

**Figure 4:**
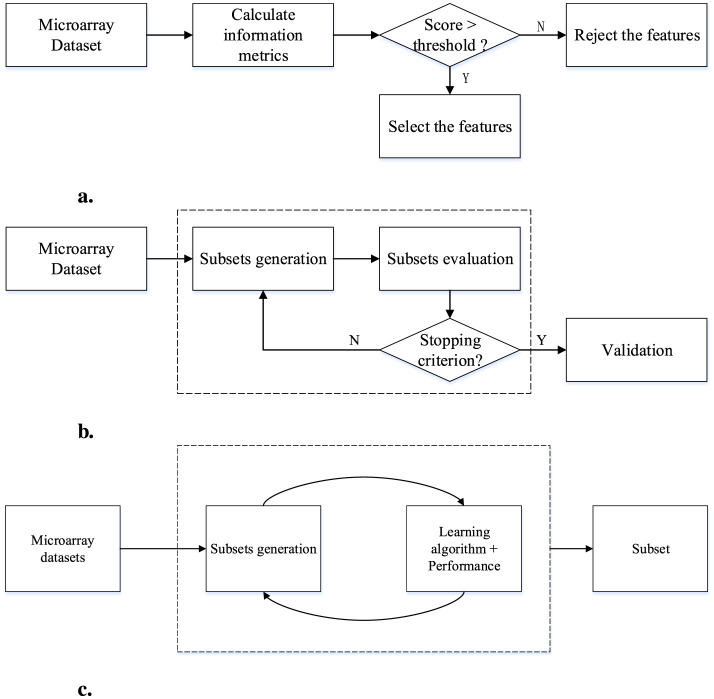
Process of different methods. **(a)** Process of filter method. **(b)** Process of wrapper method. **(c)** Process of embedded method.

Figure 4 illustrates the filter method, the wrapper method and the embedded method. The filter method in Figure 4a can ensure that the most influential features are retained, thus improving the effectiveness of the predictive model while meeting the challenges of high-dimensional datasets; the wrapper method in Figure 4b outlines the sequential steps of subset generation, evaluation, and final selection; and the embedded method in Figure 4c saves computational resources by combining feature selection with model training, and also enables feature selection to be more closely aligned with the specific goals of the model.

In addition to the main feature selection methods such as filter, wrapper and embedded methods, there are “other methods,” the main ones being hybrid methods. Hybrid methods are particularly important in microarray feature selection because of their ability to combine multiple techniques to optimize performance and mitigate limitations inherent to individual methods. Since microarray data are often rich in features, hybrid methods are more suitable for such datasets, as these methods take advantage of various selection methods to obtain subsets of features. There are multiple strategies for implementing hybrid methods, each taking advantage of different feature selection techniques to obtain more robust results. One strategy is to integrate multiple feature selection algorithms. This approach may include combining filters, wrappers, and embedding methods, with the goal of leveraging the unique strengths of each method to achieve a more comprehensive and efficient feature selection process. Another popular implementation strategy is the multi-stage feature selection method. In this approach, the selection task is divided into multiple stages, with different techniques applied at each stage. For example, filter methods can be employed in the initial stage to quickly reduce the size of the feature set. This reduced set can then be refined into a highly correlated final subset using more computationally expensive wrapper or embedded methods.

Table 3 provides a summary of the limitations and application scope of each method. When choosing an appropriate feature selection method, it is crucial to understand the characteristics of the dataset, the requirements of the current problem, and the available computing resources. It is crucial to recognize the advantages and limitations of each method, as different methods may be more suitable for different scenarios. In practical applications, a comprehensive evaluation is required on a case-by-case basis to determine the most effective feature selection method. This decision-making process ensures that the chosen method is a good fit with the goals and limitations of the study, ultimately helping to obtain more precise and reliable results in microarray data analysis.

**Table 3:** Advantages and disadvantages of different feature selection methods.

**Method**	**Advantages**	**Disadvantages**
Filter	Fast calculation speed and good time performance. Independent of the model and highly versatile. Select features through information calculation, with high interpretability.	Classification accuracy is average. Cannot completely remove redundant features. Determining an appropriate threshold can be a challenging task.
Wrapper	Ability to fully consider the interrelationships between features and find better feature subsets. Classification accuracy is high because it is closely related to the performance of a specific model. Take full advantage of the model's performance metrics to select the most relevant features.	Computationally expensive and requires training the model multiple times to evaluate performance on each feature subset. Easy to overfit, especially when the data dimension is high. Poor interpretability of selected feature subsets.
Embedded	It combines the advantages of filter and wrapper, taking into account the correlation of features and reducing computational overhead. Feature selection for a specific model can usually improve the performance of the model.	Multiple models need to be trained, so the computational overhead is relatively high. The selected features may be too dependent on the selected model and not applicable to other models.
Hybrid	Take advantage of filter, wrapper and embedded feature selection methods. It can improve the stability of feature selection and make it more general and robust. By combining multiple methods, the risk of overfitting can be reduced.	Multiple feature selection methods need to be rationally selected and tuned to ensure synergy between them. Typically, require more computational resources, as they involve the computation and integration of multiple feature selection methods.

### 2.4 Subset evaluation criteria

In microarray data analysis, evaluating feature subsets effectively is crucial for building accurate and reliable predictive models. This evaluation is conducted using a set of standards and methods known collectively as Subset Evaluation Criteria. A feature subset in this context refers to a selection of features chosen from the original set based on their potential relevance to the analysis or predictive tasks at hand. Several key metrics are commonly used to assess the performance of these feature subsets. These criteria include accuracy, recall, precision, sensitivity, and the F1 score, each serving a specific purpose in measuring different aspects of model performance: the Accuracy criterion measures the overall correctness of the predictions made by the model. It is a general indicator of how well the model performs across all classes. Recall (Sensitivity) measures the model's ability to correctly identify all positive samples. It is crucial for scenarios where missing a positive instance could have serious consequences. Precision evaluates the proportion of identified positives that are correctly predicted. High precision indicates that a model does not label negative samples as positive. F1-score metric combines precision and recall into a single metric by calculating their harmonic mean. The F1 score is particularly useful when you need to balance precision and recall, which is often the case in studies where both false positives and false negatives carry significant costs. These criteria are fundamental in assessing the quality of feature subsets and optimizing the feature selection process. They help researchers understand not just the effectiveness of the feature selection but also the potential impact of selected features on the model's ability to make accurate predictions. Each of these criteria can be quantitatively assessed using specific equations, outlined from Equations 1–4, which detail how each metric is calculated based on the true positives, false positives, true negatives, and false negatives derived from the model output.

In addition to the general evaluation criteria, the analysis of high-dimensional microarray data also relies on several specific validation methods. These methods are particularly crucial due to the challenges posed by the large number of features relative to the number of samples, a common scenario in microarray data (Alhenawi et al., 2022). These validation methods often consider two critical factors, the final subset size and the time required for the selection process. The final subset size is an important metric because it directly affects both the complexity of the model and its generalizability. A smaller subset can lead to a simpler, more interpretable model that is less likely to overfit, whereas a larger subset might capture more complex patterns at the risk of overfitting. Balancing this size is crucial for building robust predictive models. The time required for the feature selection process is another vital consideration, especially in high-dimensional data scenarios (Chen et al., 2020). Feature selection in microarray data can be computationally intensive, and the time spent selecting features can significantly impact the overall efficiency of the data analysis pipeline. Faster methods that still maintain high accuracy are preferable in scenarios where time is a constraint or when dealing with very large datasets.

The proper application of these evaluation criteria allows for a comprehensive assessment of feature subsets, guiding researchers in refining their feature selection strategies to enhance model accuracy and reliability. By using these metrics, researchers can ensure that the chosen features contribute positively to the overall performance of their models, particularly in the predictive analysis of complex biological data such as that encountered in microarray studies.


(1) $$\begin{array}{lll} Accuracy & = & \frac{TP+TN}{TP+FN+FP+TN} \end{array}$$

(2) $$\begin{array}{lll} Recall & = & \frac{TP}{TP+FN} \end{array}$$

(3) $$\begin{array}{lll} Precision & = & \frac{TP}{TP+FP} \end{array}$$

(4) $$\begin{array}{lll} F1 & = & \frac{2\times Precision\times Recall}{Precision+Recall} \end{array}$$

Where TP is true positive, which denotes the number of positive categories predicted correctly, TN is true negative, which denotes the number of negative categories predicted correctly, FP is false positive, which denotes the number of negative categories misclassified as positive, and FN is false negative, which denotes the number of positive categories misclassified as negative.

#### 2.4.1 Cross validation

Cross-validation is designed to assess the generalization ability of a model. By repeating training and evaluation on different training-validation set divisions, cross-validation helps us to reduce the model's dependence on specific data divisions and effectively prevent overfitting. In addition, cross-validation plays a key role in model selection by verifying the stability and accuracy of each configuration and selecting the optimal model.

When evaluating different models using cross-validation, the first step is to choose the appropriate method and set the relevant parameters. Taking *k*-fold cross-validation as an example, the dataset is usually divided into *k* subsets, and *k* experiments are repeated, each time one of the subsets is selected as the validation set, and the remaining *k*−1 subsets are used as the training set. This process will result in *k* validation results, and finally the mean and standard deviation of these *k* results are calculated as the overall performance metrics of the model on this dataset.

The robustness and consistency of the model can be understood by observing the average performance and standard deviation of the model across folds. Smaller standard deviations indicate that the model's performance is more stable across different data divisions; while higher average performance values indicate that the model has better generalization ability. Based on these results, the reliability of the model can be further determined and the best solution can be selected by comparing the cross-validation performance of different models or parameter combinations.

### 2.5 Summary

This section provides a summary of the basic principles of the microarray feature selection procedure. It covers important topics such as microarray data, the feature selection method, evaluation metrics for feature subsets, and the key components of the feature selection process. Understanding and implementing microarray feature selection based on these concepts is crucial for researchers to make well-informed decisions when working with microarray data.

## 3 Feature selection method on microarray

The process of feature selection is crucial in data analysis as it aims to identify the most relevant and informative features from a dataset, especially in high-dimensional data like microarray gene expression. By reducing the dimensionality of irrelevant data, feature selection enhances the efficiency and accuracy of subsequent analysis. This chapter offers a comprehensive overview of different methods of feature selection.

### 3.1 Filter feature selection method on microarray

In microarray data analysis, feature selection is a crucial step that helps identify and select genes most relevant to specific biological phenomena. The filter feature selection method is widely popular as a main strategy because it is highly efficient and easy to implement. This section explores the application of filter feature selection methods in microarray data analysis.

Nematzadeh et al. (2019) proposed a filter method employing the whale algorithm and Mutual Congestion to address this issue. They initially set the number of whales equal to the number of features and applied the whale algorithm to eliminate irrelevant features. They then ranked the remaining features using Mutual Congestion. While effective in selecting features with lower interference frequencies, the non-deterministic specification of the subset size using a threshold of 10 could benefit from an adaptive value. In another study, Li and Xu (2019) focused on obtaining effective gene expression data related to Hepatocellular Carcinoma (HCC). They utilized the Fisher score algorithm to identify characteristic HCC-related genes and performed various functional enrichment analyses. Additionally, they conducted a survival analysis to assess the relationship between selected central genes and patient survival. Addressing class imbalance, He et al. (2019) introduced the imRelief algorithm, demonstrating superior performance in handling minority sample dispersion across microarray datasets compared to various evaluation metrics.

Tavasoli et al. (2021) took measures to enhance classification accuracy. They employed data shuffling to prevent overfitting and utilized a soft-weighted ensemble mechanism with five criteria for feature selection. The study highlighted the effectiveness of combining improved algorithms and multi-mechanism soft weighting in mitigating overfitting and instability issues. However, its robustness was only tested on a limited number of benchmark datasets, requiring further verification. Furthermore, Lee et al. (2021) introduced the MB Ranking method, effectively addressing data type inconsistency in microarray datasets by leveraging the formal definition of Markov Blanket (MB) for multivariate feature ranking. This technique outperformed other ranking methods due to its inherent feature ranking advantages. In their pursuit of addressing the computational complexity linked to wrapper-based models in high-dimensional microarray datasets, Saberi-Movahed et al. (2022) introduced the Dual Regularized Unsupervised Feature Selection Based on Matrix Factorization and Minimum Redundancy (DR-FS-MFMR). This approach efficiently combines matrix factorization and subspace learning techniques to represent datasets through a matrix factorization form, enhancing the selection of more efficient features by capturing local and global correlations within the feature space. The proficiency of DR-FS-MFMR was demonstrated across nine gene expression datasets, and it was compared with nine methods using clustering accuracy and normalized mutual information. However, as feature selection was conducted via clustering, redundant features might exist within the final subset.

Overall, these studies offer a range of approaches to tackle specific challenges in feature selection in microarray datasets. Each study provides unique insights and methodologies to enhance accuracy and efficiency in selecting significant features. Filter method ranks features by calculating statistical metrics or scoring functions and does not rely on learning algorithms, the process involves calculating the statistical metrics or scoring functions for each feature, ranking the features, and selecting the top-ranked subset of features as the final result. The advantages of this method are high computational efficiency, not easy to overfitting and simplicity, which is suitable for preliminary feature screening. However, the disadvantages of the filter method are that it ignores the correlation between features, which may lead to the omission of important features, the selected subset of features may not be able to improve the model performance in some cases, and the filter method has a limited generalization ability when facing complex datasets.

### 3.2 Wrapper feature selection method on microarray

The wrapper feature selection method is a commonly used approach for finding the best feature subset using a specific algorithm. In this chapter, we will explore the use of wrapper feature selection methods in analyzing microarray data and their connection with optimization algorithms. Wrapper feature selection is closely tied to optimization algorithms. The objective of wrapper feature selection is to minimize or maximize a performance measure like classification accuracy or mean square error, thus treating it as an optimization problem. Optimization algorithms, such as genetic algorithms, simulated annealing, and particle swarm optimization, offer efficient means of optimizing wrapper feature selection methods.

In recent years, various optimization algorithms have been used for feature selection in microarrays. Almugren and Alshamlan (2019) introduced the innovative FireFly (FF) algorithm, while Chatterjee et al. (2020) improved the Social Ski Driver (SSD) algorithm by incorporating Late Acceptance Hill Climb (LAHC) to enhance its local search capabilities. They transformed the algorithm into a binary form using S-shaped and V-shaped transfer functions. To address the limited local search capabilities of the Whale Optimization Algorithm (WOA), Guha et al. (2020) introduced the embedded chaotic whale survival algorithm (ECWSA). This method introduced death and chaos mechanisms, improving the description of whale predation. Agrawal et al. (2020) proposed a feature selection method called QWOA, which modified the mutation and crossover operators applied to the quantum-inspired whale motion in WOA. Khamparia et al. (2020) developed a pioneering feature selection and classification method that utilized GA and a diverse ensemble of classifiers. They used the Bhattacharya coefficient and GA to remove noise features and derive the target feature set. Panda (2020) proposed an Elephant Search Algorithm (ESA) and Deep Learning (DL) based wrapper method for feature selection. Too and Mirjalili (2021) presented the Hyper Learning Binary Dragonfly algorithm (HLBDA) based on the Binary Dragonfly Algorithm (BDA). Abdel-Basset et al. (2021) combined the Harris Hawks Optimization algorithm (HHO) with simulated annealing (SA) to create a new feature selection approach. By using the HHO output as input for SA, they achieved a seamless integration of both algorithms. They also employed bitwise OR and bitwise AND operations to overcome limitations in population diversity that could affect HHO's performance. Das et al. (2022) introduced a novel feature selection method rooted in the Jaya optimization algorithm. By leveraging the Jaya algorithm's search technique, they streamlined the feature space by updating the weakest features. Hu et al. (2022) improved the slime mold algorithm (SMA) by employing V-shaped transfer functions to obtain binary BDFSMA. Ganesh et al. (2023) utilized the Weighted Superposition Attraction Optimization Algorithm (WSA) for microarray feature selection. There are also efficient Harmony search (HS) algorithms (Ye et al., 2023), the hybrid method developed by Bae et al. (2021) based on HS also achieved high accuracy in the colon cancer.

The wrapper method relies on a classifier to assess the performance of different subsets of features. By analyzing how well the classifier performs on a specific dataset, the wrapper method can choose the best subset of features to improve the model's performance. Some commonly used classifiers in this method are KNN, DT, RF, SVM, and others. KNN is especially popular among researchers. For example, Chatterjee et al. (2020), Guha et al. (2020), Too and Mirjalili (2021), Abdel-Basset et al. (2021), Hu et al. (2022), and Ganesh et al. (2023) used SSD, WOA, BDA, HHO, SMA, and WSA respectively in combination with the KNN classifier to achieve feature selection in microarray datasets. Other commonly used classifiers include SVM and deep learning (DL). Almugren and Alshamlan (2019) used a combination of FireFly and SVM, while Panda (2020) used ESA and DL for microarray dataset classification.

There are also approaches that involve multiple classifiers. For instance, Khamparia et al. (2020) developed a convolutional neural network with multiple classifiers to create a multi-level ensemble model for diagnosing neuromuscular samples. The ensemble method, based on deep convolutional neural networks, showed superior accuracy in disease diagnosis and prediction compared to other classifiers. In addition to combining multiple classifiers, there are cases where the same algorithm is used to test the classification effect of different classifiers. Agrawal et al. (2020) and Das et al. (2022) used multiple classifiers, such as KNN, LDC, SVM, C4.5, and RT, to compare their classification effects.

Table 4 provides a comprehensive summary of the methodologies, algorithms, and their performance in feature selection and classification across various studies.

**Table 4:** Summary of wrapper methods.

**References**	**Algorithms**	**Classifier**	**Dataset**	**Evaluation criteria**	**Key findings**
Almugren and Alshamlan (2019)	FF	SVM	Leukemia2, SRBCT Lung, Leukemia1, Colon	ACC, Num-F	Comparing the advantages and disadvantages of wrapper method and hybrid method. The algorithm has good stability on different datasets.
Chatterjee et al. (2020)	SSD	KNN	Breastcancer, BreastEW, Exactly, Exactly2, HeartEW, M-of-n, DLBCL, SRBCT, Leukemia2	ACC, Num-F	SSD was first used in feature selection. It is verified that the effect of the S-type transfer function is slightly better than that of the V-type.
Guha et al. (2020)	ECWSA	KNN	Breast, BreastEW, Exactly, Exactly2, HeartEW, AMLGSE2191, Colon, DLBCL, Leukaemia, Prostate, MLL, SRBCT	ACC, Num-F	Improved the weak local search ability of the whale algorithm. Due to the mechanism of local search, the computational complexity will increase.
Khamparia et al. (2020)	Bhattach arya-GA	KNN, DT, LDA, QDA, RF, SVM	E-GEOD-3307 are divided into two datasets	ACC, computational time	Using multi-level ensemble methods to use different model results as input to deep networks. Another attempt of neural network in feature selection.
Agrawal et al. (2020)	QWOA	K-NN, LDC, SVM, and C4.5	GLI-85, LA_BRA180 9Tumor, GCM	ACC, AUC, *F*-value, Num-F	Use a clustering step for high-dimensional datasets to reduce feature input before feature selection. Compared with the classic algorithm WOA, the performance of the quantum algorithm QWOA is better and has been verified.
Panda (2020),	ESA	DL	Prostate, Leukemia, Colon, DLBCL, Ovarian, Breast, CNS, Lung-Harvard, MLL, SRBCT	ACC, Num-F, running time	Use One way ANOVA and *Post hoc* Tukey HSD Test to verify algorithm suitability. Verified the effectiveness of using DL models as classifiers.
Too and Mirjalili (2021)	HLBDA	KNN	TOX_171, Colon, Leukemia	ACC, Num-F	Compared with many methods, HLBDA obtained the best fitness and average fitness. The classification accuracy results of HLBDA in high-dimensional datasets are higher than other methods.
Abdel-Basset et al. (2021)	HHO and SA	KNN	Colon, Leukemia	ACC, Num-F, F-value, running time	Using bitwise OR and bitwise AND operations to overcome LO and low population diversity. In high-dimensional datasets, the fitness value is not the best of the comparison methods.
Das et al. (2022)	Jaya	NB, KNN, LDA and RT	Brest Cancer, SPECTF heart, Colon, Leukemia	ACC, Num-F	Get the final subset by removing features. Comparing the performance of NB, KNN, LDA and RT four classifiers.
Hu et al. (2022)	SMA	KNN	Leukemia, Brain, Lung_Cancer, Prostate, CNS, 11Tumors, 9Tumors, Brain2, DLBCL, Leukemia1, Leukemia2, Tumors_14	ACC, Num-F, *F*-value, running time	After continuous space verification, apply it to MA feature selection. This method has the disadvantage of long running time.
Ganesh et al. (2023)	WSA	KNN	Ovarian, Colon	ACC, Num-F	WSA was first used in feature selection. WSA is only compared with the original version of other algorithms.

Wrapper method microarray feature selection evaluates and selects a subset of features by using the performance of a learning algorithm, and the process involves starting with an initial set of features, gradually adding or removing features, and evaluating the effect of different subsets of features based on the performance metrics of the learning algorithm, and ultimately selecting the subset of features with the best performance as the result. The advantage of this approach lies in the direct optimization objective, which can better optimize the performance of the final model by directly using the performance of the learning algorithm to evaluate the feature subset. In addition, the wrapper method is flexible and can be combined with multiple learning algorithms to adapt to different data and tasks, and is usually capable of selecting a relatively small, but superior performance feature subset. However, the drawbacks of this method are the high computational cost and the need to train the learning algorithms multiple times to evaluate the performance of different feature subsets, which is computationally expensive. In addition, due to multiple evaluations on the training data, the wrapper method is susceptible to overfitting, which may reduce the generalization ability of the model on test data, and as the number of features increases, evaluating all the possible combinations of features becomes infeasible.

### 3.3 Embedded feature selection method on microarray

The essence of embedded feature selection lies in its integration with the model training process. This means that the selection of features is inherently tied to the learning algorithm. This approach allows for the concurrent optimization of both the model parameters and the feature subset, with the aim of enhancing the model's efficacy on both the training and validation datasets. In this section, we delve into contemporary embedded feature selection techniques. We examine their foundational principles, procedural frameworks, and their respective merits and limitations when applied to microarray data analysis. Furthermore, we showcase the practical utility of these methods through their application to real-world datasets. We analyze their performance across various contexts and highlight their comparative strengths.

Sun et al. (2019) addressed issues of data distribution in the error-correcting output coding (ECOC) algorithm by leveraging Data Complexity theory. Their algorithm optimized ECOC encoding matrices and consistently outperformed state-of-the-art algorithms across microarray datasets. Lopez-Rincon et al. (2019) proposed an integrated feature selection strategy that utilized multiple techniques and classifiers. Their approach aimed to discover robust miRNA signatures and demonstrated high classification accuracy across diverse datasets and platforms. Climente-González et al. (2019) developed Block HSIC Lasso, a feature selection method adept at handling ultra-high-dimensional data. This method showcased enhanced performance with larger datasets and required fewer features to achieve comparable classification accuracy to other methods. Tang et al. (2019) tackled non-IID features through latent representation learning and graph-based manifold regularization (LRLMR). Despite not excelling in one dataset, this innovative unsupervised feature selection method exhibited robust intrinsic data structure characterization in microarray datasets. Kang et al. (2019) proposed rL-GenSVM for high-dimensional tumor datasets. This method combined Relaxed Lasso for feature selection with GenSVM as the classifier. The approach effectively selected and classified features in tumor datasets. Jeon and Oh (2020) introduced the Hybrid-RFE ensemble algorithm, which amalgamated SVM-RFE, RF-RFE, and GBM-RFE methods. This method, validated on UCI and NCBI gene expression datasets, showcased improved performance over single RFE methods. This improvement was mainly due to weight summation, which significantly reduced the number of features while enhancing accuracy. In their pursuit of minimal yet informative gene combinations, Peng et al. (2021) introduced the multi-layer iterative feature selection method, MGREF. Their GA-REF algorithm, a fusion of Genetic Algorithm (GA) and Recursive Feature Elimination (REF), operated in a multi-layer fashion, dividing datasets and proceeding through three distinct stages. While effectively retaining optimal features, this method preserved a slightly larger feature set than existing selection methods. The method proposed by Hamla and Ghanem (2024) selects the top ranked features obtained from the Fisher score to provide a candidate subset for the embedding stage. Then Support Vector Machine Recursive Feature Elimination is utilized and applied to the candidate subset to find the best subset. To achieve better classification accuracy of Lasso in DNA microarray data classification, Vatankhah and Momenzadeh (2024) used a method to automatically find the optimal regularization parameters. Results on four commonly used datasets demonstrate the effectiveness of the method.

Embedded feature selection methods can tightly integrate feature selection and model parameter optimization with the model training process, thereby improving model performance on training and validation datasets. This approach allows automatic selection of the most relevant feature subsets within the framework of a learning algorithm, avoiding a separate feature selection step. The advantage is that feature selection is embedded in model training, which can process data efficiently. It can also optimize model parameters and feature subsets at the same time, improving the overall performance of the model. Feature selection and model training are performed simultaneously, which reduces the process of manual intervention and improves the overall performance. However, this approach relies on specific learning algorithms that increase the complexity and training time of the model, and some embedded methods may only be applicable to specific types of data or tasks and may not be as effective as specialized feature selection methods in some cases.

From the distribution of publication years, it is evident that most articles concerning embedded feature selection are concentrated in the year 2019. This trend may be correlated with the robustness of embedding methods and the advancement of alternative techniques. The robustness of embedding methods hinges upon the chosen machine learning models, the selection of an inappropriate model for a specific dataset or problem may result in unstable feature selection outcomes. Among alternative methods, hybrid methods are predominantly utilized. These methods effectively enhance model generalization by amalgamating the outcomes of various feature selection techniques. By integrating multiple approaches, hybrid methods better capture genuine patterns within the data and mitigate the risk of overfitting, thereby enhancing the predictive capacity of models on novel samples.

The studies examined various embedded feature selection methods, each offering unique strategies to optimize feature subsets within datasets. Table 5 is a comprehensive summary table that encapsulates the key methodologies, algorithms, and their performance in feature selection and classification across various studies. These embedded feature selection methodologies catered to diverse dataset complexities. They offered strategies to optimize feature subsets efficiently while addressing specific challenges in data distribution and dimensionality.

**Table 5:** Summary of embedded methods.

**References**	**Key algorithm and classifier**	**Key findings**
Sun et al. (2019)	DC theory, Gaussian SVM, NB	Consistently superior performance among ECOC algorithms.
Lopez-Rincon et al. (2019)	Multiple feature selection and many classifiers	High classification accuracy, cross-platform applicability.
Climente-González et al. (2019)	Block HSIC Lasso, Random Forest	Better performance with larger datasets with fewer features required.
Tang et al. (2019)	LRLMR, KNN	propose a robust unsupervised feature selection method with latent representation learning and graph embedding.
Kang et al. (2019)	Relaxed Lasso, GenSVM	Use regularization term to avoid overfitting and achieves better accuracy.
Jeon and Oh (2020)	SVM-RFE, RF-RFE, GBM-RFE	Enhance performance over single RFE methods.
Peng et al. (2021)	GA-REF, *t*-test, MIC	A multi-layer recursive feature elimination method based on the embedded integer coding genetic algorithm MGRFE.

### 3.4 Hybrid feature selection method on microarray

In addition to classic methods, a variety of alternative strategies have emerged in the field of feature selection. Among these, hybrid methods have become one of the most popular methods, commonly involving a combination of filter and wrapper methods. When filter and wrapper methods are used together for microarray feature selection, the filter method initially eliminates irrelevant features quickly based on specific criteria or statistical metrics. Subsequently, the wrapper method selects features that significantly impact prediction accuracy under the guidance of model performance. Intelligent optimization algorithms are often employed for efficient subset search, and classifiers are typically used for evaluation. For instance, Shukla et al. (2019b) introduced the TLBOSA method, combining Teaching Learning-based Optimization and Simulated Annealing algorithms, utilizing SVM as a fitness function. Alanni et al. (2019) employed Information Gain (IG) and Gene Expression Programming for initial feature selection, followed by SVM-based fitness function for further refinement. Loey et al. (2020) proposed an intelligent decision support system utilizing IG for initial gene selection and Gray Wolf Optimization algorithm (GWO) for feature reduction, coupled with SVM for classification. Moreover, Alomari et al. (2021) introduced rMRMR-MGWO, combining mRMR and GWO methods, leveraging SVM for classification. Houssein et al. (2021) utilized IG in conjunction with Barnacles Mating Optimizer Algorithm (BMO) and SVM for feature selection. Mahesh et al. (2024) developed a new method for predicting leukemia microarray gene data based on a new technique of hybrid ant lion mutant colony optimization as well as PSO. Dabba et al. (2021b) proposed MIM-mMFA, employing MIN-MAX, Maximum Mutual Information (MIM), and a modified Moth Flame Algorithm for feature selection alongside SVM. Additionally, Dabba et al. (2021a) introduced another approach where mRMR is used in the first stage, and in the second stage, a quantum moth flame optimization algorithm (QMFOA) and SVM are employed to achieve similar effects.

Random Forest (RF), k-Nearest Neighbors (KNN), and Naive Bayes (NB) are also widely used. For example, Shukla et al. (2019c) proposed a feature selection framework, and the specific implementation steps are called Filter-Wrapper Feature Subset Selection (FWFSS). This hybrid method uses a conditional mutual information maximization-based filter method and GA algorithm-based wrapper method to enhance the overall classification performance, using the NB classifier as the fitness function during the wrapper method. This hybrid method outperforms the compared many existing filter algorithms in both classification accuracy and optimal number of features. Ali and Saeed (2023) also developed a hybrid method based on GA. Pashaei and Pashaei (2019) incorporated RF into their approach. Initially, they employed RF ranking to remove noise and redundant features. Subsequently, they applied the Intelligent Dynamic Genetic Algorithm (IDGA) and a RF-based wrapper method for Microarray feature selection. Tatwani and Kumar (2019) introduced a method termed Master-slave Genetic Algorithms (GAs) for feature selection. Their approach begins with an initial preprocessing stage utilizing IG to eliminate redundant features. Subsequently, employing the Master-slave GA and RF for feature selection. Additionally, it needs more comparison with other algorithms, necessitating further research to ascertain its effectiveness comprehensively. Alhenawi et al. (2023) developed a hybrid method based on improved intelligent water drop algorithm and filter method. Sahu and Dash (2024) developed a method based on Jaya algorithm and IG. Sucharita et al. (2024) applied moth-flame optimization and extreme learning machine for Microarray feature selection. Dash et al. (2022) employed statistical measures to select the top 100 features. They improved the Shuffled Frog Leaping Algorithm (SFLA) by adjusting the frog jumping step size and combined it with KNN for microarray feature selection. Experimental comparison results on binary classification datasets indicate certain advantages of this method, demonstrating its effectiveness.

There are also some studies that use different classifiers for comparison. Gangavarapu and Patil (2019) proposed a hybrid greedy ensemble approach optimized using the GA to reduce the dimensionality of high-dimensional biomedical datasets. This method uses different information measures in the filter stage and compares the efficiency of KNN, DT and RF classifiers in the wrapper stage. Shukla et al. (2019a) introduced various methods of methodology. Initially, they utilized Conditional Mutual Information Maximization (CMIM) for the primary feature selection stage. Subsequently, the Binary Genetic Algorithm (BGA) served as the fitness evaluator for the features. Furthermore, classifiers such as KNN, SVM, DT and RF were employed to compute the subset's fitness value. Shukla et al. (2020) take advantage of the advantages of teaching learning-based algorithm (TLBO) and gravitational search algorithm (GSA) algorithms to develop a new high-search efficiency algorithm, TLBOGSA, and introduce a new encoding strategy to convert its continuous search space into a binary search space. Before using TLBOGSA for feature selection, mRMR is first used to select a feature subset, and then the wrapper method based on TLBOGSA is used for feature selection, they compared the effects of four classifiers, SVM, KNN, DT and NB, and finally confirmed that NB classifier is the most effective.

Some other microarray feature selection methods are hybrids of the two methods. To capture the interaction of features and solve the classification problem of data imbalance, Hosseini and Moattar (2019) proposed a hybrid feature selection method called mutual information and Monte Carlo-based feature selection (MIMCFS). The technique is divided into two stages: mutual information to select main features and the Monte Carlo tree search technique to eliminate redundant features. However, in this method, some parameters are set based on an empirical basis. Finding a better method for setting these parameters may lead to better experimental results. Kilicarslan et al. (2020) employed the ReliefF and Stacked AutoEncoder (SAE) methods for dimensionality reduction. Subsequently, they utilized SVM and Convolutional Neural Networks (CNN) for classification. The dimensionality reduction and classification techniques were combined pairwise to validate the accuracy of feature selection. Jain and Singh (2021) proposed a fast, general-purposed, influential hybrid feature selection approach with an adaptive classification method for chronic disease datasets that can enhance the classifier's efficiency and decrease computation cost and time. This approach outperforms the traditional SVM classifier regarding all significant performance measures and shows outstanding results. The critical aspect of the approach is the selection of an appropriate threshold for selecting relevant features from the dataset. Dash (2021) combined the Harmony Search and Pareto Optimization methods to develop a new hybrid MA feature selection method. The first 100 features are generated using the adaptive harmony search based gene selection (AHSGS) method in the first stage. In the second stage, a bi-objective Pareto optimization method was employed to reduce the gene subset further through evaluation using four different classifiers, including KNN, NB, ANN, and SVM. It was found that when paired with the SVM classifier, it outperforms other classifiers. Zare et al. (2023) achieved the maximum relevance criterion by integrating a supervised Laplace eigenmap and a matrix, and then minimized the redundancy between the selected features by applying a Pearson correlation coefficient.

Furthermore, some studies combine multiple methods for feature selection. Prabhakar and Lee (2020) proposed a tri-level feature selection method to boost prostate cancer classification accuracy. Initially, discrete wavelet transformation reduces feature count. Subsequent steps involve employing various selection methods on the simplified feature set. Experimentation highlighted the best accuracy achieved by combining the MA feature selection method, Signal Noise Ratio (SNR), and Whale Optimization Algorithm (WOA), utilizing an Artificial Neural Network (ANN) as the classifier. Mandal et al. (2021) introduced a Tri-Stage Wrapper-Filter Feature Selection Framework for Disease Classification. In the initial stage, multiple filter methods (MI, CS, RFF, XV) and classification algorithms (KNN, SVM, NB) are combined to ensure high accuracy for each feature regardless of the filter method used. In the second stage, correlation analysis (PCC) removes highly correlated features from the top k features obtained in the first stage, aiming for a maximally informative yet minimally redundant subset. Following these stages, XGBoost further refines the feature set. Lastly, a WOA-based wrapper approach finalizes the optimal feature subset. This innovative framework effectively merges wrapper and filter methods, enhancing classification accuracy while reducing computational complexity. Overall, this approach provides a novel method for disease classification, potentially improving diagnostic and therapeutic outcomes.

Each study presented in this collection highlights innovative strategies that incorporate a combination of feature selection techniques. This underscores the substantial importance of employing hybrid methods to improve accuracy and efficiency in microarray data analysis. In the landscape of microarray data analysis, the evolution of hybrid feature selection methods has proven instrumental in surmounting challenges inherent to high-dimensional datasets. Through a fusion of filter, wrapper, and ensemble techniques, these methodologies have navigated the complexities of feature selection, attaining heightened accuracy, reduced redundancy, and improved computational efficiency. While each approach brings unique insights and strengths, their convergence into hybrid methodologies reflects a pivotal stride in advancing the accuracy and applicability of microarray data analysis.

Hybrid method microarray feature selection combines multiple feature selection techniques and classifiers to improve the effectiveness of feature selection and model performance by combining the advantages of different methods. The strength of this method lies in its versatility and robustness. By combining multiple methods, it is possible to capture data features more comprehensively, improve the robustness of feature selection, and typically achieve higher classification accuracy than a single method. In addition, hybrid method feature selection has the flexibility to adapt to specific problems and data characteristics by flexibly adjusting the combined methods. However, its drawbacks include increased complexity, the need to evaluate multiple combinations, high computational cost and time-consuming, and the difficulty of optimization, which requires careful adjustment and optimization of the combinations of individual methods and classifiers, which is more difficult. For a comprehensive overview of Hybrid methods articles, refer to Table 6.

**Table 6:** Summary of hybrid methods.

**References**	**Key algorithm and classifier**	**Evaluation criteria**
Shukla et al. (2019b)	CFS-TLBOSA-SVM	ACC, Num-F
Alanni et al. (2019)	IG-GEP-SVM	ACC, Num-F, Running time
Loey et al. (2020)	IG-GWO-SVM	ACC, Robustness
Alomari et al. (2021)	mRMR-GWO-SVM	ACC
Houssein et al. (2021)	IG-BMO-SVM	ACC
Dabba et al. (2021a)	MIN-MAX/MIM-mMFA-SVM	ACC, Num-F
Dabba et al. (2021a)	mRMR-QMFOA-SVM	ACC, Num-F
Shukla et al. (2019c)	MIM-GA-NB	ACC, Num-F
Pashaei and Pashaei (2019)	RF-IDGA-RF	ACC
Tatwani and Kumar (2019)	IG-GAs-RF	ACC
Dash et al. (2022)	Statistical measures, SFLA-KNN	ACC
Gangavarapu and Patil (2019)	IG/PCC/mRMR/oneR/Correlation-GA-KNN/DT/RF	ACC, Robustness
Shukla et al. (2019a)	CMIM-BGA-SVM/KNN/NB/DT	ACC, Num-F
Shukla et al. (2020)	mRMR-TLBOGAS-NB/SVM/KNN/DT	ACC
Kilicarslan et al. (2020)	ReliefF-SAE-SVM/CNN	ACC
Jain and Singh (2021)	Adaptive classification method	ACC
Dash (2021)	HS and Pareto Optimization	ACC
Prabhakar and Lee (2020)	Tri-level approach for classification	ACC
Mandal et al. (2021)	Tri-Stage Wrapper-Filter Framework	ACC, running time

### 3.5 Other feature selection method on microarray

In addition to these methods, Multi-objective algorithms also play an important role in feature selection, especially when competing objectives need to be balanced. The prediction accuracy of the model and the size of the feature subset are two key objectives in the feature selection task. Traditional single-objective optimization methods usually focus on a single objective, such as maximizing the accuracy of the model, which may result in selecting too large a subset of features, increasing the computational cost and complexity of the model. The other extreme is to oversimplify the feature subset, which reduces the computational cost but may also impair the predictive performance of the model. Multi-objective algorithms are able to generate a set of Pareto-optimal solutions by simultaneously optimizing multiple objectives, each of which represents the equilibrium point where one objective cannot be further improved without degrading the other. In the process of feature selection, accuracy and feature subset size are often the two most critical and competing objectives. Multi-objective algorithms are able to consider these two factors simultaneously, providing researchers with a set of different solutions. By analyzing the Pareto frontier, researchers can achieve a better balance by choosing the most suitable feature subset among these solutions based on specific application scenarios and requirements. For instance, Cao et al. (2019) proposed a feature selection method that considers classification error, number of features, and redundancy among features based on the Distributed Parallel Collaborative Coevolutionary Multi-Objective Large-Scale Evolutionary Algorithm. To reduce calculation time, they introduced feature number constraints respectively to reduce feature input. A distributed parallel strategy is adopted to parallelize the evolution process. Adopt sample-level parallelism strategies to parallelize the testing process. Qiu (2019) developed an innovative feature selection method, MSPSO, utilizing a multi-swarm PSO algorithm. This approach subdivided the population into sub-swarms to maintain diversity, with an elite learning strategy facilitating information exchange among these sub-swarms. The experiments highlighted MSPSO's superiority over traditional PSO-based methods and popular filters in feature subset size and classification accuracy. Zhang et al. (2020) proposed a multi-objective feature selection algorithm based on binary differential evolution incorporating self-learning strategies. This algorithm embedded novel operators like binary mutation and One-bit Purifying Search to balance local exploitation and global exploration, showcasing improved performance in reducing initial feature sets' complexity. Baliarsingh et al. (2020) presented a framework called C-HMOSHSSA for gene selection in cancer classification using multi-objective meta-heuristic and machine learning methods. The proposed framework utilizes the multi-objective spotted hyena optimizer and slap swarm algorithm for gene selection, with the goal of finding a minimum subset of genes while maximizing classification accuracy. The authors conducted experiments using seven different microarray datasets to evaluate the performance of the proposed technique and compared it with existing state-of-the-art techniques. Aljarah et al. (2020) used two operators, a dynamic time-varying strategy and local fittest solutions, to improve the performance of multi-objective SSA for feature selection and used the S-shaped function to convert the improved SSA into MODSSA-Ibest, which can achieve feature selection. It can achieve faster convergence speed while avoiding local optimal solutions. Judging from the performance on both microarray datasets, features were reduced by more than 40%, and significant results were also achieved in terms of average error rate and g-mean. Dhal and Azad (2021) present a multi-objective hybrid binary version of the FS approach based on two evolutionary approaches, PSO and GWO. The approach can efficiently learn from a smaller number of samples and high-dimensional data and simultaneously considers two objectives: classification error rate and the number of features. The paper introduces a novel concept, population factor, for generating the population and a modified version of the velocity update equation based on Newton's second law of motion. The search space is divided into two phases, global and local search, and the efficacy of the method is evaluated using benchmark high-dimensional datasets. Han et al. (2021) proposed a new feature selection method based on an adaptive strategy multi-objective particle swarm optimization algorithm called MOPSO-ASFS. MOPSO-ASFS uses the PBI decomposition method to adaptively provide different penalty values for each weight vector so that more optimal solutions are retained on the Pareto front. Zhou et al. (2021) proposed an evolutionary multi-objective optimization framework of discretization-based feature selection for classification. Many heuristic search methods can be used in this framework; they take PSO, for example, as the search method; to obtain the Pareto solutions, a flexible cut-point PSO is introduced to help better explore relevant subsets of features. Moslemi and Ahmadian (2023) developed a new feature selection method based on rank constrained and dual regularized nonnegative matrix factorization, which outperforms the latest unsupervised feature selection techniques in multiple mediums in terms of clustering accuracy and normalized mutual information. Analogously, Samareh-Jahani et al. (2024) developed a low-redundancy unsupervised feature selection method based on data structure learning and feature orthogonalization, which first uses QR decomposition to obtain an orthogonal representation of the feature space, and then determines the distance between the feature set and the orthogonal set obtained from the original features based on a matrix decomposition. Also, Saberi-Movahed et al. (2024) proposed a deep non-negative matrix factorization method by combining global and local structures that preserves both global and local structures in the data space. Furthermore, regularization terms that promote sparsity by exploiting the notion of inner product are applied to represent matrices of lower dimensions as a way to preserve the underlying data structure while discarding less important features. Sheikhpour et al. (2025) proposed a feature selection method expressed in the form of trace ratios, which utilizes the discriminative information of labeled data to maximize class separability, as well as the hypergraph Laplace operator to capture geometric structure and higher-order relationships in labeled and unlabeled data. Lv et al. (2021) proposed an innovative framework, SFAM, that combines adaptive global structure learning and stream shape learning with the aim of improving the effectiveness of semi-supervised multi-label feature selection. The framework overcomes the limitations of existing methods in dealing with label correlation by utilizing both local and global data structures. The authors also develop an efficient iterative optimization algorithm to address the non-smooth objective function of the model.

The multi-objective method to feature selection finds the best subset of features by simultaneously optimizing multiple objectives (for example, classification accuracy and number of features). It is characterized by multi-objective optimization, which provides more comprehensive feature selection results and efficiently handles conflicts and trade-offs between different objectives through the optimization algorithm. This approach is widely applicable and can be applied to a variety of data types and tasks with strong adaptability. The advantages of the multi-objective approach include the ability to optimize on multiple performance metrics at the same time, providing more comprehensive and effective results than the single-objective approach. It is flexible and applicable to complex tasks that need to balance multiple performance requirements, and can adjust the optimization objectives according to different needs. In addition, through search methods such as evolutionary algorithms, the multi-objective approach may find the global optimal or near-optimal subset of features. However, the computational complexity of this approach is high, especially when dealing with high-dimensional data, which requires larger computational resources and time. Implementation complexity is also a major challenge, requiring a deep understanding of the principles and methods of multi-objective optimization. In addition, multi-objective optimization produces a potentially large set of solutions (Pareto front), and selecting the best solution and interpreting its significance may be more difficult.

Besides, there are also some less commonly used methods. Zhang et al. (2021) proposed a feature selection method based on information-theoretic lower bounds of feature inner correlations for high-dimensional data. The authors introduce two lower bounds for feature redundancy and complementarity, which have simple forms and are closer to the optima than existing lower bounds used by some state-of-the-art information-theoretic methods. They then propose a simple and effective feature selection method based on these lower bounds and verify its effectiveness with a wide range of real-world datasets. Xie et al. (2024) proposed a graph neural network-based feature selection algorithm with a classification model to achieve feature selection. They use a multidimensional graph to represent interactions between genes, utilize link prediction techniques to enrich existing graph structure relationships, and use a multidimensional node evaluator and a spectral clustering-based supernode discovery algorithm to achieve initial screening of nodes. Subsequently, we further screen the nodes using downsampling-based hierarchical graph pooling techniques to achieve feature selection and build classification models.

### 3.6 Summary of different methods

This section will present some summaries on microarray feature selection, including the number of articles on different methods in recent years, the classifiers used by different methods and the classification accuracies of various methods.

#### 3.6.1 Comparison of different methods

Filter method is a model-independent feature selection technique that performs feature selection by calculating the correlation or amount of information between the features and the target variable. The main advantage of this method is that it is computationally efficient and suitable for large-scale datasets because it does not require model training for each feature combination. Meanwhile, the filter method is highly interpretable, and the feature selection process is intuitive and easy to understand and implement. However, the disadvantage of the filtering method is that it tends to ignore the interrelationships between features and relies only on the correlation of individual features with the target variable, which may leave out certain important features. This neglect may result in compromising the performance of the model in cases where there are important interactions between features, thus limiting its predictive power in practical applications.

Wrapper method is better able to capture the complex relationships between features by training the model while selecting features and considering the interactions between features. This method typically provides superior feature selection results because it evaluates the effectiveness of feature combinations based on the performance of the model. However, the computational overhead of the wrapper method is high because the model needs to be trained for each feature selection, especially on large datasets, which can significantly increase the consumption of computational resources. In addition, repeatedly evaluating the model performance may lead to overfitting. The reason is that it evaluates the performance of feature combinations by repeatedly training the model. During the feature selection process, the wrapper method constantly adjusts the feature subset based on the performance of the training set, and this high-frequency model evaluation may result in a model that overfits the noise and features of the training data, thus performing well on the training set but having reduced generalization ability on new, unseen data. Especially in the case of a small sample size of the training set, the model's over-reliance on a specific combination of features may make the selected features not representative, thus decreasing the prediction accuracy in real-world applications.

Embedded method combines the advantages of the filter and wrapper methods by automatically selecting features during the model training process. This method takes into account the interactions between features and is relatively efficient because feature selection is synchronized with model training. The embedded method is able to reduce the computational complexity and usually yields better feature selection results. However, its drawback is that it strongly depends on the selected model, this is because it integrates the feature selection process directly into the training of the model, making the assessment of the importance of features dependent on the model algorithm used. Different models, use different criteria to assess the importance of features, which means that the feature selection results can vary from model to model. In addition, the effectiveness of the embedded method is closely linked to the generalization ability of the model, and if the selected model performs well on a specific dataset but poorly on other datasets, the results of feature selection may also lack generalization. This dependency not only affects feature selection, but also requires that when faced with a new problem or dataset, feature selection may need to be redone to accommodate the new model configuration. As well, embedded methods usually require tuning the hyperparameters of the model to optimize performance, and different hyperparameter settings can also lead to variations in the feature selection results. Therefore, when using embedding methods, researchers and practitioners need to have a deep understanding of the characteristics and behaviors of the models used to ensure that the selected features can effectively support model learning and prediction, and to avoid performance degradation or improper selection due to model dependency.

Hybrid method combines the advantages of the filter and wrapper methods by first performing initial feature screening through the filter method to quickly exclude irrelevant features, and then performing refined selection through the wrapper method. The advantage of this approach is that it increases both the efficiency of feature selection and the accuracy of the final feature set. However, the hybrid approach is more complex to implement and requires coordinating the implementation of the two methods, which can lead to misconfigurations. In addition, the use of wrapper methods may still consume significant computational resources on large datasets, despite the fact that the initial screening reduces the number of features. The complex implementation process may lead to irrational feature selection, which affects the performance of the model, especially when the features are poorly selected, which may result in the model not being able to learn the structure of the data efficiently.

#### 3.6.2 Method selection guidelines

In real-world biomedical applications, the choice of an appropriate feature selection method strongly depends on the dataset characteristics and practical constraints. For example, filter-based methods are usually preferred when dealing with high and small sample datasets due to their simplicity and scalability. Wrapper methods, while usually yielding higher accuracy, may not be suitable for large datasets due to their high computational cost. Embedded methods provide a compromise by integrating model training and feature selection, making them more popular in scenarios where classifier performance is critical. Hybrid methods are especially valuable when both selection quality and computational feasibility are required. These practical considerations are crucial when applying feature selection to tasks such as personalized medicine or early cancer diagnosis.

When selecting an appropriate feature selection method for microarray datasets, which typically exhibit high dimensionality, low sample size, and class imbalance, it is essential to make targeted decisions based on the specific characteristics of the data. For datasets with extremely small sample sizes and extremely high feature dimensions, such as Leukemia, Colon, and Prostate, the Filter method is recommended. This method is computationally efficient, relatively robust to small samples, and can quickly eliminate a large number of redundant features. Additionally, the Filter method does not depend on specific learners and is suitable as a pre-screening step in the first phase to reduce the difficulty of subsequent modeling.

In datasets with significant class imbalance, such as Leukemia1, MLL, and CNS, Hybrid methods or Wrapper methods with class-aware mechanisms perform more stably. Especially under feature score bias caused by class imbalance, Hybrid methods can effectively mitigate bias by combining independent scoring with model feedback. For multi-class datasets, such as SRBCT, Lymphoma and Leukemia2, it is important to consider the method's support for multi-class discrimination capabilities. In such tasks, embedded methods like LASSO and tree models are more suitable. These methods can dynamically adjust feature importance during training based on the objective function and effectively account for inter-class differences, adapting to multi-class structures.

In summary, different feature selection methods have their own advantages on different types of microarray datasets. Filter methods are suitable for datasets with high feature redundancy and severe small sample problems; Hybrid and Wrapper methods demonstrate high adaptability when dealing with class imbalance, and Embedded methods are suitable for multi-class classification or tasks with high requirements for feature interdependencies. By reasonably combining different methods, more stable and interpretable results can be achieved in various data scenarios.

#### 3.6.3 Number of papers with different methods

**Figure 5:**
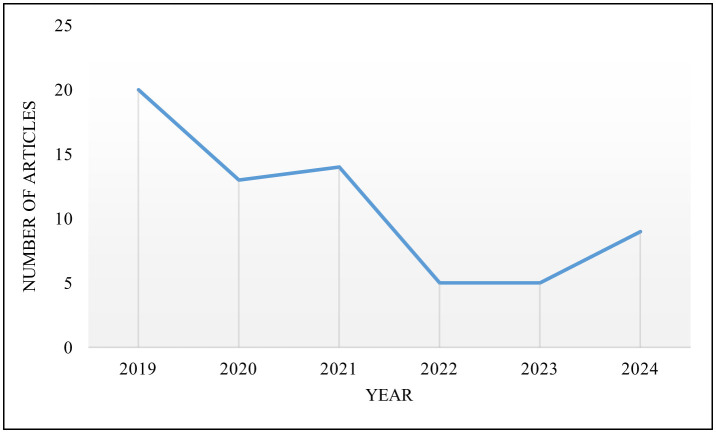
Number of articles every year.

The number of papers for every year can be found in Figure 5. The research on feature selection for microarray data has experienced a trend of first decline and then rise in the number of articles, mainly due to the fact that initially researchers focused on simple feature selection methods, while with the maturity of the technology and the rise of deep learning and integration methods, the research has gradually shifted to more complex techniques, which led to a decline in the number of studies on traditional methods. However, in recent years, with the improvement of computational power and the increase of data complexity, the research on feature selection has become active again, and the number of related literatures has risen rapidly, especially driven by new technologies. This change is attributed to technology iteration, data complexity, and cross-fertilization between fields such as bioinformatics and computer science. Studying feature selection can not only significantly improve model performance, reduce computational resource consumption and risk of overfitting, but also help to extract biologically significant and important features, facilitate the understanding of disease mechanisms, and promote the development of new algorithms and models. Therefore, the study of feature selection is of great significance for the progress and innovation in the field of microarray data analysis.

In conclusion, while hybrid methods dominate the landscape of microarray feature selection, challenges remain in achieving comprehensive and interpretable results. Continued research efforts focused on improving classification accuracy, reducing dimensionality, and enhancing interpretability are essential to unlock the full potential of microarray feature selection in biomedical applications.

#### 3.6.4 Classification results

**Figure 6:**
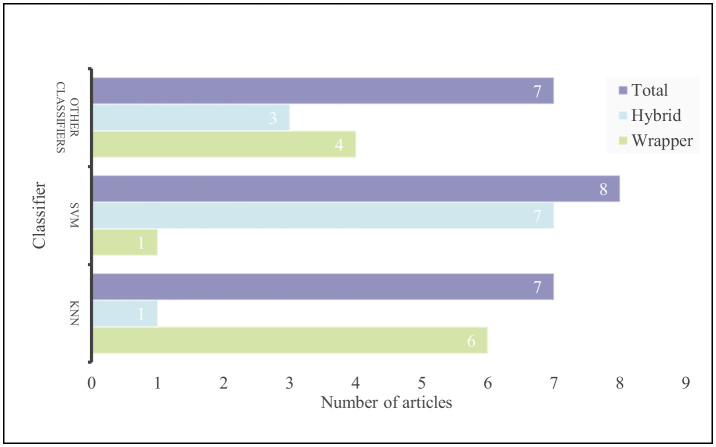
Number of articles using different classifiers.

Classifiers play a pivotal role in feature selection by serving as the core component for evaluating and selecting feature subsets. They aid in identifying which features are most conducive to predicting the target variable by training on the training set and assessing their performance. The performance of classifiers frequently serves as a criterion for selecting feature subsets, and they are also utilized to guide the optimization of feature subsets. This ensures that the chosen feature subset enhances performance of the model and generalization capabilities. We have summarized the frequency with which different methods employ various classifiers, as depicted in Figure 6. The figure illustrates that KNN and SVM are frequently employed as classifiers, likely due to their robustness and generalization capabilities, making them effective in handling high-dimensional data. The KNN classifier is known for its simplicity and ease of implementation. It operates by identifying the K instances in the training dataset that are closest to the new sample and making predictions based on their majority class. This method is particularly suitable for processing nonlinear data and scenarios with numerous outliers. On the other hand, SVM stands out as a powerful supervised learning algorithm that excels in separating different categories of data by identifying a hyperplane that maximizes the classification margin. It is adept at handling both linearly separable and inseparable problems, and can be extended to address nonlinear challenges through kernel techniques. Given these attributes, KNN and SVM have become staples in microarray feature selection. They assist researchers in extracting valuable insights from intricate gene expression data, consequently enhancing prediction accuracy and the biological interpretability of models. Their versatility and effectiveness make them indispensable tools in the pursuit of understanding and leveraging genetic information for various applications in biomedicine and beyond.

The classification results obtained from microarray datasets hold significant implications for disease diagnosis, biomarker discovery, drug development, understanding disease mechanisms, and advancing personalized medicine. Accurate classification of microarray data unveils the relationship between gene expression patterns and biological states, furnishing a scientific foundation for medical decision-making, fostering precision medicine development, and facilitating profound biomedical research endeavors. Thus, ensuring the precision of classification outcomes for microarray datasets is imperative for research and clinical applications in related domains. This study compiled data on the classification accuracy of various methods applied to microarray feature selection, utilizing commonly employed datasets. The statistical classification accuracy of microarray feature selection methods is delineated in Tables 7, 8. In both tables, “–” means that the feature selection method is not tested on this dataset. Furthermore, in addition to assessing classification accuracy, some methods use the dimensionality of the selected feature subset as an evaluation criterion for the feature selection process. The entries highlighted in bold in the tables represent the methods that produce the highest performance on the corresponding dataset. These tables are valuable resources for researchers and practitioners to gain insight into the efficacy of different feature selection methods in accurately classifying microarray data. By utilizing this information, informed decisions can be made regarding the selection and implementation of feature selection techniques to advance the field of microarray data analysis and its diverse applications in biomedical and other fields.

**Table 7:** Classification accuracy on binary class datasets.

**References**	**Breast**	**Ovarian**	**CNS**	**Prostate**	**DLBCL**	**Leukemia**	**Colon cancer**
Tavasoli et al. (2021)	98.2 (7)	–	–	**100 (10)**	–	100 (6)	99.3 (7)
Saberi-Movahed et al. (2022)	–	–	68.10 (40)	68.62 (10)	91.59 (70)	31.94 (10)	88.06 (40)
Nematzadeh et al. (2019)	–	–	80.00 (–)	–	–	–	90.00 (–)
Abdel-Basset et al. (2021)	–	–	–	–	–	93.30 (–)	84.60 (–)
Das et al. (2022)	99.12 (–)	–	–	–	–	96.36 (–)	76.59 (–)
Ganesh et al. (2023)	–	100 (–)	–	–	–	–	100 (–)
Almugren and Alshamlan (2019)	–	–	–	–	–	99.50 (11)	93.50 (19)
Panda (2020)	73.43 (4)	99.21 (14,771)	56.67 (5,603)	88.24 (8,334)	91.49 (2,310)	100 (4,667)	79.03 (1,429)
Guha et al. (2020)	–	–	–	96.30 (9)	**100 (24)**	**100 (4)**	**100 (30)**
Peng et al. (2021)	–	–	**100 (7)**	98.10 (4)	**100 (3)**	**100 (2)**	98.50 (6)
Shukla et al. (2019c)	–	–	–	95.32 (20)	90.01 (20)	–	90.15 (18)
Shukla et al. (2019c)	–	–	–	99.13 (8)	99.52 (11)	–	99.01 (12)
Jain and Singh (2021)	–	89.33 (5,432)	–	83.33 (6,333)	–	80.95 (3,394)	71.43 (812)
Pirgazi et al. (2019)	88.17 (10.2)	–	95.64 (6.7)	94.18 (7.8)	99.21 (6.8)	99.62 (5.2)	94.72 (5.3)
Dabba et al. (2021a)	**100 (150)**	100 (200)	100 (150)	100 (80)	100 (100)	100 (10)	100 (80)
Dabba et al. (2021a)	77.53 (27.73)	99.37 (20.60)	100 (31.27)	99.87 (32.60)	–	100 (36.47)	100 (30.67)
Dash (2021)	93.00 (–)	–	–	–	88.00 (–)	96 (–)	74 (–)
Shukla et al. (2020)	–	–	–	98.42 (7)	99.62 (17)	–	98.87 (16)
Alomari et al. (2021)	–	**100 (3.56)**	99.38 (17.46)	–	–	–	95.86 (9.8)
Shukla et al. (2019c)	–	–	–	96.15 (21)	89.91 (25)	–	85.24 (23)
Qiu (2019)	–	–	–	–	–	71.24 (–)	81.63 (–)
Aljarah et al. (2020)	–	–	–	–	–	98.90 (–)	97.90 (–)
Baliarsingh et al. (2020)	88.88 (20)	100 (150)	–	–	–	99.21 (40)	95.06 (100)
Han et al. (2021)	–	–	–	–	85.13 (–)	–	78.56 (–)

Bold values indicate the best accuracy among all methods.

**Table 8:** Classification accuracy on multi-class datasets.

**References**	**MLL**	**Leukemia 1**	**Brain tumor 2**	**11 Tumor**	**Brain tumor 1**	**9 Tumor**	**Leukemia 2**	**Lung cancer (Harvard)**	**SRBCT**
Lee et al. (2021)	98.61 (130)	–	–	–	–	–	–	96.55 (110)	100 (10)
Panda (2020)	80.56 (12,392)	–	–	–	–	–	–	94.10 (8,056)	93.98 (1,540)
Almugren and Alshamlan (2019)	–	–	–	–	–	–	92.58 (19)	–	97.50 (12)
Guha et al. (2020)	100 (8)	–	–	–	–	–	–	–	100 (30)
Peng et al. (2021)	**100 (3)**	–	–		–	–	–	–	100 (5)
Shukla et al. (2019c)	–	95.35 (12)	–	92.23 (13)	96.98 (12)	73.51 (11)	99.57 (7)	99.87 (10)	99.91 (5)
Shukla et al. (2019c)	–	–	–	–	–	–	–	98.18 (22)	89.31 (17)
Shukla et al. (2020)	–	94.15 (16)	–	93.04 (13)	96.92 (15)	70.88 (12)	98.84 (12)	99.61 (13)	99.17 (11)
Alomari et al. (2021)	100 (8.4)	–	–	–	–	–	–	97.91 (15.8)	100 (12.3)
Houssein et al. (2021)	–	–	–	–	–	–	**100 (6)**	–	**100 (4)**
Dabba et al. (2021a)	100 (130)	–	100 (150)	100 (130)	100 (150)	100 (150)	100 (50)	100 (130)	100 (80)
Dabba et al. (2021a)	–	–	100 (34.73)	**100 (40.73)**	**100 (35.40)**	**100 (39.27)**	100 (35.53)	100 (26.60)	99.44 (28.27)
Alanni et al. (2019)	–	100 (–)	99.90 (–)	99.88 (–)	99.80 (–)	98.88 (–)	100 (–)	100 (–)	100 (–)
Shukla et al. (2019c)	–	–	–	–	–	–	–	98.32 (24)	89.02 (19)
Zhou et al. (2021)	–	94.83 (45.2)	76.92 (172.7)	88.16 (473.6)	74.83 (133.4)	50.02 (64.4)	97.30 (171.4)	84.18 (454.4)	99.63 (52.2)
Dhal and Azad (2021)	–	–	**100 (10)**	97.85 (52)	97.95 (37)	86.67 (37)	–	99.83 (38)	100 (10)

Bold values indicate the best accuracy among all methods.

This chapter explores different feature selection methods for microarray data analysis, including filter, wrapper, embedded, hybrid and other methods, evaluating their strengths and weaknesses. Hybrid methods, combining various techniques, have emerged as powerful tools, enhancing model performance and interpretability. With technology advancements and increasing data volume, hybrid methods are expected to play a key role in future research, offering adaptable solutions to address evolving challenges in microarray data analysis and biomedical research.

## 4 Microarray feature selection application

Microarray feature selection technology holds a central position in disease classification and diagnosis. By scrutinizing patient gene expression data through feature selection, it markedly enhances early diagnostic precision, identifies disease subtypes, discovers biomarkers, and predicts drug sensitivity. The successful application of this high-throughput analysis method, notably in cancer research, has significantly influenced both medical research and clinical practice.

In real-world biomedical research, feature selection methods are often chosen with more than just classification accuracy concerns. Researchers must also balance the computational efficiency of the algorithm, the cost of implementation, and the interpretability of the results. For example, in time-sensitive clinical environments such as cancer subtype prediction or diagnostic screening, the ability to obtain results quickly may be more important than small improvements in accuracy. Therefore, an algorithm that can provide acceptable accuracy in a shorter period of time may be preferred to some complex but computationally expensive methods.

Data distribution is also a key consideration in real-world applications, especially in scenarios with small sample sizes or severe category imbalances. For example, in disease prediction tasks, the number of positive cases is usually small. In such cases, distance-based feature selection methods may perform poorly due to neighborhood structure bias. In contrast, some hybrid or embedded methods that incorporate category prior information or regularization strategies tend to have better stability. Therefore, in specific applications, in addition to the method category, its robustness to sample bias is also an important criterion for method selection.

Therefore, the adoption of feature selection methods in the real application often requires a trade-off between algorithm complexity, selection accuracy and scalability. This trade-off is especially critical when translating computational research results into clinical practice, where clinical environments often have practical constraints on time, interpretability, and compatibility with downstream analysis tools. Taking these factors into account can help in choosing feature selection methods that have both theoretical strengths and practical needs.

Several advanced methodologies have been proposed for microarray feature selection and classification. Rochayani et al. (2020) introduced a two-stage method employing the Lasso regularization method followed by Classification and Regression Trees for further refinement and classification. Xie et al. (2022b) proposed a feature selection algorithm and classification model grounded in graph neural networks, overcoming existing method limitations by enriching graph structural relationships via link prediction techniques. Wu et al. (2022) utilized XGBoost followed by the gray wolf algorithm to pinpoint the optimal gene subset for cancer classification. Wang et al. (2020) devised a novel feature selection approach within the ensemble learning framework, corroborating its robustness through multiple aggregation methods. Zare et al. (2023) advocated a supervised feature selection approach based on manifold learning, integrating Supervised Laplacian eigenmaps and matrices for comprehensive feature selection. Prajapati et al. (2023a) employed ant colony optimization in tandem with logistic regression, decision tree, and random forest for exhaustive feature selection and classification accuracy comparison. Prajapati et al. (2023c) used a genetic algorithm for feature selection in combination with classification algorithms such as logistic regression, decision tree, and random forest to detect cancer, tumors and various other diseases. Sahu and Dash (2024) proposed a hybrid FS model based on the Jaya optimization algorithm and information gain, which verified the effectiveness of IG technology in feature selection. Additionally, there are many cases where microarray feature selection has been used in disease subtype diagnosis. Mehrabani et al. (2022) used microarray gene expression data from 72 patients with acute myeloid leukemia (AML) and lymphoblastic leukemia (ALL), and the RF and SVM classifiers correctly classified all AML and ALL samples.

Disease subtypes play a significant role in disease classification and treatment choice. Understanding disease subtypes supports personalized medicine and tailored treatment strategies. Maulik et al. (2013) demonstrated the effectiveness of feature selection and transductive SVM in predicting cancer subtypes. Roberts et al. (2018) distinguished clinically relevant cancer subtypes using a differential variance classifier, with combined methods yielding superior results. Wang et al. (2023b) validated the efficacy of feature selection and Bayesian networks in identifying protein biomarkers for cancer subtypes.

Biomarkers serve as crucial indicators in disease diagnosis, monitoring, and assessing treatment effectiveness. Trevizan and Recamonde-Mendoza (2021) proposed Ensemble Feature Selection for identifying potential breast cancer biomarkers. Colombelli et al. (2022) developed a hybrid ensemble feature selection design to enhance the reproducibility of genomic biomarker discovery. Xie et al. (2022a) introduced a novel biomarker selection method, demonstrating its effectiveness in feature reduction and classification accuracy improvement. Alzubaidi et al. (2022) addressed challenges in breast cancer staging by developing a deep learning-based feature extraction module for identifying robust biomarkers. Ge (2023) proposed FSRL for identifying potential biomarkers for various high-mortality cancers, demonstrating superior classification accuracy and computational efficiency.

Drug sensitivity prediction employs various methods and techniques to anticipate an individual's response to specific medications, leveraging their biological characteristics, genomic information, or other biomarkers. This predictive approach serves the goal of personalized medicine in the medical field, striving to maximize drug treatment efficacy while minimizing adverse reactions. Microarray technology plays a crucial role in this endeavor by collecting gene expression data from individual samples and scrutinizing the correlation between this data and drug responses. For instance, Chen and Sun (2017) devised a novel method for high-dimensional dual-layer feature selection, utilizing a set of response variables that share a standard set of predictive variables. Simulation results indicate heightened sensitivity and specificity compared to existing methods. Meanwhile, Ahmed et al. (2020) described a network-based approach for identifying features in drug response prediction. They employed a gene co-expression network to pinpoint representative features and proposed a graph neural network model integrating gene network information for outcome prediction. Koras et al. (2020) introduced a prior-knowledge-driven feature selection method grounded in drug targets, target pathways, and gene expression features. Validation underscored the importance of selecting appropriate feature selection strategies, particularly for drugs targeting specific genes, pathways, or affecting general mechanisms such as immune response and DNA replication. These models show promise in guiding treatment design. Ataei et al. (2021) initially employed gene fuzzy score and principal component analysis to reduce data dimensions, followed by SVM classification of sensitive and resistant data samples. Subsequent Wilcoxon Rank Sum tests determined differentially expressed genes, contributing to the understanding of drug sensitivity mechanisms. Yang et al. (2022) proposed a cancer drug sensitivity prediction model based on multi-omics data constructed using stacked ensemble learning methods. Through functional annotation and enrichment analysis of feature genes, they elucidated potential resistance mechanisms of tumors to sorafenib, substantiating the model's interpretability from a biological perspective. This model holds promise in guiding clinical drug usage.

Given the diverse application scenarios outlined above, selecting an appropriate feature selection strategy must be tailored to the specific goals and data characteristics of each task. In microarray data analysis, the objective of feature selection is not singular but closely tied to the problem being addressed. For disease subtype classification, classification accuracy and generalization capability are critical, especially as the model needs to capture subtle yet important expression differences. Hence, wrapper or embedded methods are commonly adopted, as they can adaptively optimize feature subsets based on model performance feedback. These approaches are better suited for capturing nonlinear relationships and dealing with the challenges of high dimensionality and limited sample sizes. Some studies have further enhanced performance by incorporating transfer learning and graph-based structures to model complex biological dependencies.

In contrast, biomarker discovery emphasizes the stability, reproducibility, and biological interpretability of selected features. In such cases, filter methods are often favored due to their reduced dependency on specific classifiers and increased robustness across datasets. Recently, ensemble strategies and multi-criteria fusion techniques have gained popularity. These combine multiple scoring metrics or selection algorithms to ensure that the resulting biomarkers are both statistically significant and biologically meaningful.

For drug sensitivity prediction, the goals extend beyond classification accuracy to include interpretability and generalizability across diverse biological conditions. Since drug responses often involve intricate molecular mechanisms and multi-omics interactions, this domain frequently employs network-based analysis, embedding methods, and automated feature engineering techniques. These are often combined with ensemble learning or multi-layer integration models to enhance predictive performance. Furthermore, dimensionality reduction techniques such as PCA are commonly used during preprocessing, followed by supervised feature evaluation, to maintain both model robustness and biological interpretability.

**Figure 7:**
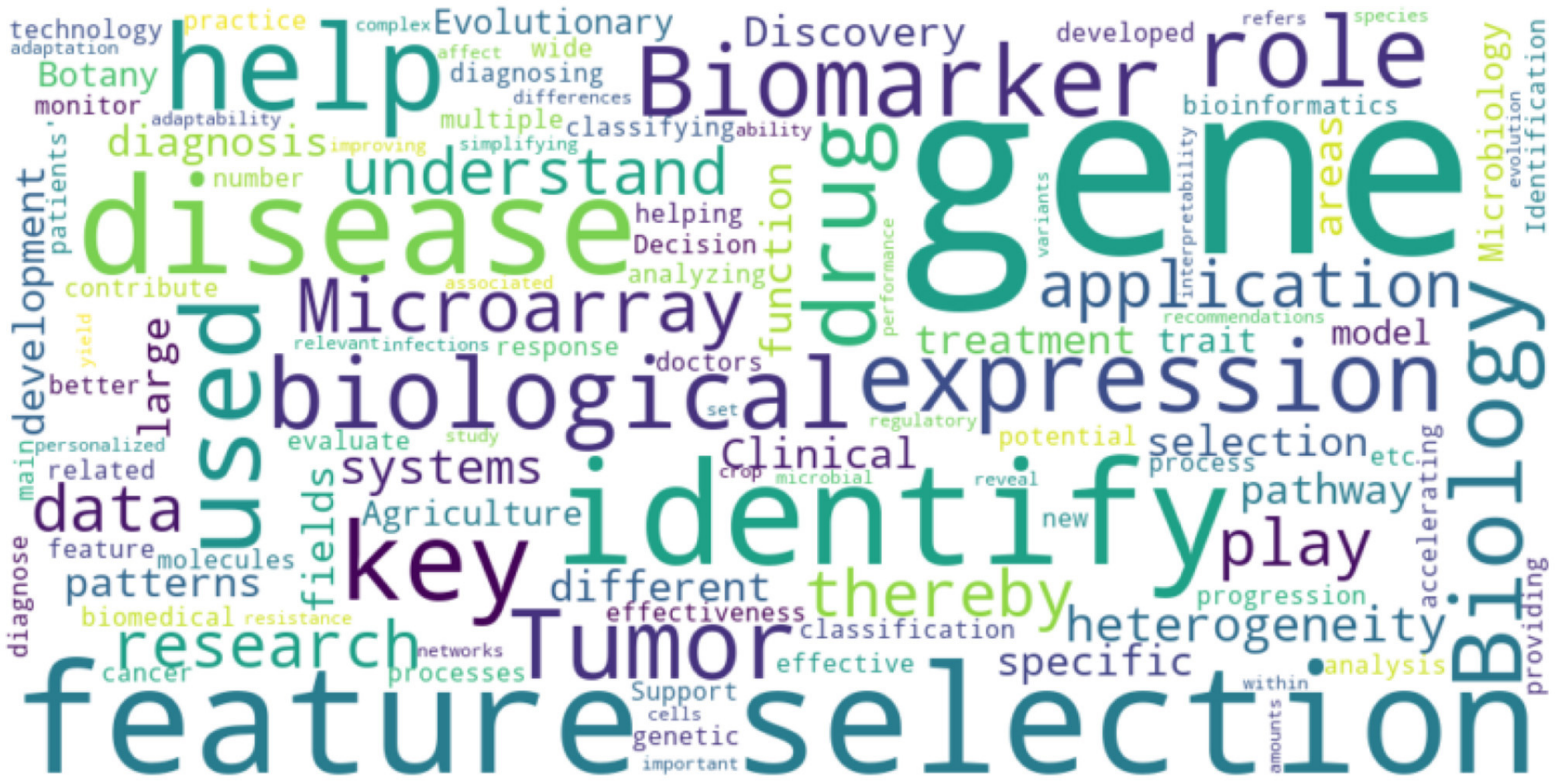
Word cloud of application areas.

In conclusion, microarray feature selection is integral to disease diagnosis, biomarker discovery, and drug sensitivity prediction. Its application in disease subtype diagnosis, biomarker discovery, and drug sensitivity prediction underscores its significance in advancing personalized medicine and improving treatment outcomes. Figure 7 illustrates a schematic diagram of a word cloud generated by the application of microarray feature selection in various fields. The prominent keywords include “gene,” “feature selection,” “identify,” “disease,” and “biology.” This visualization underscores the significance of microarray feature selection in bioinformatics research. It plays a pivotal role in selecting valuable information from complex gene expression data, thereby advancing biomedical research and contributing to the understanding and treatment of diseases.

The application trends in microarray feature selection are primarily characterized by the integration of deep learning technologies, the incorporation of multi-modal data integration, standardization and annotation, the pursuit of interpretability and biological relevance, the expansion into clinical applications, and the emphasis on privacy protection and security. These trends highlight the rapid evolution and shifting research focus within the field of microarray feature selection, foreshadowing both opportunities and challenges ahead. As technology advances and data volumes grow, these trends are expected to continue evolving, bringing forth more innovations and breakthroughs in the field of microarray data analysis.

## 5 Summary and future work

This study systematically reviews the literature on microarray feature selection and explore its significance in both academic and practical fields. By integrating existing studies, we aim to help readers gain a comprehensive understanding of the overall development of microarray feature selection, including the strengths and weaknesses of different approaches and their applicability scenarios. We identify gaps in current research and point out areas that have not yet been fully explored to provide clear directions for subsequent research, thus stimulating the academic community to explore new techniques and applications.

In addition, this review provides a comprehensive assessment of various feature selection methods, aiming to provide researchers with theoretical foundations and practical guidance in selecting methods suitable for their specific research questions, in order to promote the optimization of existing techniques and the development of new methods. We emphasize the importance of fostering communication and collaboration between multiple fields, including bioinformatics, computer science, and statistics, to help researchers draw on best practices from other disciplines to further advance microarray analysis techniques.

Finally, by emphasizing the importance of feature selection in real-world applications such as personalized medicine, cancer diagnosis, and drug discovery, we hope to enhance the understanding of the value of these techniques among industry practitioners, and thus promote their implementation in practice. In summary, this review not only provides theoretical support for the academic community, but also provides practical guidance for the practical field, significantly contributing to the overall improvement of microarray data analysis techniques.

With the development of technology, the field of microarray feature selection is facing unprecedented innovation opportunities, and its future development will focus on the dual breakthroughs of technological innovation and practical applications. At the technical level, the deep integration of deep learning and feature selection will become an important breakthrough. By building a deep feature selection framework with adaptive capability, researchers can automatically capture high-order nonlinear feature interactions in the data and significantly improve the accuracy and efficiency of feature selection. Meanwhile, the optimization innovation of integrated learning methods will promote the establishment of multi-algorithm collaborative selection mechanism, which will realize the synergistic enhancement of the stability and generalization of the feature selection results through intelligent weighted fusion and dynamic voting strategies. It should be noted that, while performing performance breakthroughs, interpretability has become a key bottleneck in the development of this field. The “black-box” nature of the current deep feature selection model severely restricts its application in clinical practice, so there is an urgent need to develop new algorithms with both high performance and interpretability, as well as a framework for evaluating the importance of features by integrating causal reasoning, to provide a transparent and traceable scientific basis for biomedical decision-making.

In application expansion, microarray feature selection will play a greater role in the future in the fields of precision medicine, drug discovery and multi-omics analysis. As research shifts from static classification to dynamic prediction and mechanism exploration, feature selection will help model disease processes and develop individualized treatment strategies. The fusion of multi-omics data will promote the systematic understanding of complex disease mechanisms, while in drug development, feature selection will accelerate target identification and drug response prediction. In addition, the technology will be expanded to systems biology, environmental monitoring and other emerging fields to support the in-depth analysis of complex biological systems and ecological factors. In the future, microarray feature selection is expected to become an important tool for data-driven knowledge discovery, promoting the transformation of life science research into intelligent and systematic.

It is worth noting that in recent years, with the rapid development of reinforcement learning (RL) and large language models (LLMs), their potential in microarray feature selection has gradually attracted attention. Reinforcement learning models feature selection as a sequential decision-making process, where an agent dynamically adjusts the selected feature subset based on feedback. This not only improves selection efficiency and has strong generalization capabilities. For example, the reinforcement learning-based automated feature selection framework proposed by Liu et al. (2021) demonstrates better robustness and selection stability on multiple high-dimensional datasets. Fan et al. (2020)'s AutoFS design integrates diversity reward mechanisms and interactive reinforcement learning strategies, enhancing interpretability while maintaining performance. Additionally, multi-agent collaborative selection methods have also achieved outstanding results in feature subspace exploration (Liu et al., 2019).

On the other hand, the combination of large language models and structured data analysis also shows great potential. The CAAFE framework proposed by Hollmann et al. (2023) integrates LLM with tabular predictors to achieve an integrated process of feature construction, model guidance, and interpretation. This framework can combine biological background knowledge with natural language instructions in practical applications, assisting researchers in efficiently identifying biologically meaningful features from complex gene expression data. The integration of these technologies not only provides smarter and more automated tools for feature selection, which also expands new possibilities for cross-modal data analysis and human-machine collaborative modeling.

While this review systematically compares and synthesizes experimental results reported in previous literature, we acknowledge that this study has not yet conducted new empirical benchmarking experiments. As this paper is a literature-focused review, its scope and focus dictate that we primarily rely on existing experimental results to draw comparative conclusions. However, we recognize the importance of standardized, unified benchmarking across different datasets and methods and plan to incorporate such comparative assessments in future research. By conducting research under consistent experimental conditions, we aim to provide a more objective assessment of the strengths and weaknesses of each method, thereby offering deeper empirical insights into microarray feature selection techniques.

In the future, microarray feature selection technology will continue to develop under the dual-wheel drive of algorithm innovation and application expansion. On the one hand, with the introduction of cutting-edge technologies such as interpretable AI and causal inference, the feature selection process will be more transparent and reliable; on the other hand, its in-depth application in the fields of precision medicine and drug discovery will continue to promote the transformation of biomedical research into a new paradigm of data-driven and knowledge discovery. These advances will significantly enhance the analytical value of microarray data, and will revolutionize human health research and clinical practice.
